# Rice developmental stages modulate rhizosphere bacteria and archaea co-occurrence and sensitivity to long-term inorganic fertilization in a West African Sahelian agro-ecosystem

**DOI:** 10.1186/s40793-023-00500-1

**Published:** 2023-05-17

**Authors:** Donald Tchouomo Dondjou, Abdala Gamby Diedhiou, Daouda Mbodj, Marie-Thérèse Mofini, Sarah Pignoly, Cheikh Ndiaye, Issa Diedhiou, Komi Assigbetse, Baboucarr Manneh, Laurent Laplaze, Aboubacry Kane

**Affiliations:** 1 Département de Biologie Végétale, Faculté des Sciences et Techniques, Université Cheikh Anta Diop (UCAD), Dakar, Sénégal; 2 Laboratoire Mixte International Adaptation des Plantes et Microorganismes associés aux Stress Environnementaux (LAPSE), Centre de recherche de Bel-Air, Dakar, Sénégal; 3 Laboratoire Commun de Microbiologie (LCM), Centre de Recherche de Bel-Air, Dakar, Sénégal; 4 Centre d’Excellence Africain en Agriculture pour la Sécurité Alimentaire et Nutritionnelle (CEA‑AGRISAN), UCAD, Dakar, Sénégal; 5 Centre d’Etude Régional pour l’Amélioration de l’Adaptation à la Sécheresse (CERAAS), Institut Sénégalais de Recherches Agricoles (ISRA), Route de Khombole, Thiès, Sénégal; 6 Africa Rice Center (AfricaRice), Saint-Louis, Senegal; 7 DIADE, Université de Montpellier, IRD, CIRAD, Montpellier, France; 8 Laboratoire Mixte International Intensification Écologique Des Sols Cultivés en Afrique de L’Ouest (IESOL), Dakar, Sénégal; 9 Eco&Sols, Université de Montpellier, IRD, CIRAD, INRAE, Institut Agro, Montpellier, France; 10 Centre d’Excellence Africain « Environnement, Sociétés » (CEA-AGIR), UCAD, Santé, Dakar, Sénégal

**Keywords:** Metabarcoding, 16S rRNA gene, Fertilization, Inter-kingdom network

## Abstract

**Background:**

Rhizosphere microbial communities are important components of the soil-plant continuum in paddy field ecosystems. These rhizosphere communities contribute to nutrient cycling and rice productivity. The use of fertilizers is a common agricultural practice in rice paddy fields. However, the long-term impact of the fertilizers usage on the rhizosphere microbial communities at different rice developmental stages remains poorly investigated. Here, we examined the effects of long-term (27 years) N and NPK-fertilization on bacterial and archaeal community inhabiting the rice rhizosphere at three developmental stages (tillering, panicle initiation and booting) in the Senegal River Delta.

**Results:**

We found that the effect of long-term inorganic fertilization on rhizosphere microbial communities varied with the rice developmental stage, and between microbial communities in their response to N and NPK-fertilization. The microbial communities inhabiting the rice rhizosphere at panicle initiation appear to be more sensitive to long-term inorganic fertilization than those at tillering and booting stages. However, the effect of developmental stage on microbial sensitivity to long-term inorganic fertilization was more pronounced for bacterial than archaeal community. Furthermore, our data reveal dynamics of bacteria and archaea co-occurrence patterns in the rice rhizosphere, with differentiated bacterial and archaeal pivotal roles in the microbial inter-kingdom networks across developmental stages.

**Conclusions:**

Our study brings new insights on rhizosphere bacteria and archaea co-occurrence and the long-term inorganic fertilization impact on these communities across developmental stages in field-grown rice. It would help in developing strategies for the successful manipulation of microbial communities to improve rice yields.

**Supplementary Information:**

The online version contains supplementary material available at 10.1186/s40793-023-00500-1.

## Background

The global demand for food is expected to rise by at least 60% by 2050 [1, 2]. Rice is a major staple food accounting for over 20% of global calorie intake and feeding over 50% of the global population [3–5]. It is grown in more than a hundred countries on over 10% of global cropland, with an annual production of more than 700 million tons [6, 7]. To meet the rising demand from a growing world population, a 40% increase in rice production must be achieved by the end of 2030 [8], on limited and increasingly degraded arable lands and in the context of global climate change [5, 9, 10].

Rice farmers largely rely on fertilizer application to maintain or increase yields [11, 12]. However, excessive use of inorganic fertilizers can adversely affect soil health [13, 14], and leads to environmental problems such as soil, water and air pollution, and greenhouse gas emission [15, 16]. Harnessing the potential of symbiotic and non-symbiotic beneficial soil microbes has been proposed as an alternative to inorganic fertilizers [17–19]. Indeed, some soil microbes positively influence plant health and productivity directly by providing nutrients and growth-stimulating factors, by enhancing tolerance to pathogens and abiotic stresses, or indirectly by regulating nutrient availability in soil through the processes of organic matter decomposition and solubilization or by stabilizing soil aggregates [20–22]. Through their multiple functions, soil microbes play a pivotal role in ecosystem services and biodiversity conservation [23–25].

Several studies have shown that long-term agricultural management practices profoundly affect soil physicochemical properties, and thereby, alter microbial community composition, structure, and function [26–28]. For instance, long-term mineral fertilization often results in significant increase in the soil microbial biomass in cropping systems [29], with positive or negative effects on soil microbial enzyme activities of nutrient cycling [30, 31]. It has also been shown that long-term mineral fertilization in a paddy soil alters community structure of ammonia-oxidizing bacteria rather than archaea [32].

On the other hand, the composition and structure of the microbial communities inhabiting the rhizosphere (root-soil interface, [33, 34]) can be shaped by the plant genotype and the plant developmental stage [35–37]. Indeed, the root morphology, the root exudates composition and the plant host immune system, which differ among genome types and developmental stages, are among the specific traits by which plants modulate the rhizosphere microbial communities [38–40].

In West Africa, the effects of inorganic nitrogen (N), phosphorous (P) and potassium (K) fertilizer management practices on rice yield have been widely studied in different agroecosystems by agricultural research organizations for many years [41]. In the Senegal River Delta, Haefele et al. [42] revealed that long-term (26 years or 52 seasons of rice cultivation) application of inorganic N, P and K fertilizers has resulted in significant increases in grain yield of rice, while significant effects on total nutrient concentrations in the soil were only detected for P. However, the impact of long-term application of N, P and K fertilizers on microbial communities inhabiting the rice rhizosphere in West African Sahelian agroecosystems remains unknown. Despite the effect of plant age [43], as well as those of inorganic fertilization [44, 45] on microbial communities inhabiting the rice rhizosphere were separately documented elsewhere, we still lack a comprehensive understanding on the sensitivity to inorganic fertilization of rhizosphere microbial communities across developmental stages under paddy field conditions. The identification of critical rice developmental stages during which the rhizosphere microbial communities are particularly affected by inorganic fertilization, as well as the fertilization sensitive microbial taxa would help in developing strategies for the successful manipulation of microbial communities to improve rice yields. Indeed, it could help to understand the inconsistent persistence of certain inoculants in the rhizosphere and to identify the right time of application of certain inoculants in relation to rice developmental stages and inorganic fertilization [43, 46, 47].

Hence, the main objectives of the present study were to assess (1) the effects of long-term (27 years) N and NPK-fertilization on bacterial and archaeal community inhabiting the rice rhizosphere in the Senegal River Delta, (2) the sensitivity to inorganic fertilization of rhizosphere bacteria and archaea across rice developmental stages and (3) the inter-kingdom co-occurrence patterns of rhizosphere bacteria and archaea across rice developmental stages.

## Methods

### Site description

Rhizosphere soil samples analyzed in this study were collected from a long-term fertility experiment (LTFE) conducted over a period of 27 years (1991–2017) at the AfricaRice Sahel research station in Ndiaye (16° 11’ N, 16° 15’W) located close to the coast (about 40 km inland) in the Senegal River Delta (Senegal, West Africa). The long-term fertility experiment included six fertilizer treatments (consisting of different combinations of N, P and K fertilizers) laid out in a randomized complete block design and rice was grown for two seasons per year (for a detailed description see Bado et al. [48]).

The local climate is a typical Sahelian climate with a long dry period from October to June and a short-wet season from July to September [49]. The highest average temperatures are recorded in April-May and the lowest in December-January. The soil is an orthothionic Gleysol, containing 40–54% clay (smectite and kaolinite) with average permeability of 2.8 mm.d^− 1^. Soil salinity is high due to the occurrence of marine salt deposits in the sub-soil [50]. The average precipitation is 177 mm during the wet season and 7.5 mm during the hot dry season [49].

### Experimental design and rhizosphere soil sampling

Rhizosphere soil samples were collected during the hot dry season in 2017, from three replicates of three fertilizer treatments of the LTFE: unfertilized plots; N-fertilized plots with the recommended dose of N fertilizer without P and K (120 kg N/ha, 0 kg P/ha, 0 kg K/ha); and NPK-fertilized plots with the recommended dose of NPK fertilizer (120 kg N/ha, 26 kg P/ha and 50 kg K/ha). Each plot measured 25 m^2^ (5 × 5 m) and contained rice seedlings of the variety Sahel 108 (IR 13240-108-2-2-3). The plots were separated by small dikes (30 cm high) and maintained in irrigated conditions. During the rice cultivation, fertilizers were applied as follows: 40% N, 100% P and 100% K were applied at tillering, 40% N at panicle initiation and the remaining 20% N at booting stage. Rhizosphere soil samples were collected at those three developmental stages at which the fertilizers were applied.

At each rice developmental stage (tillering, panicle initiation and booting), rhizosphere soil samples were taken two times (one day before and two days after fertilizer application). Hence, 6 sampling time-points (3 developmental stages x 2 time-points) were obtained for each of the three fertilizer treatments. At each sampling time-point, the entire root system of 3 individual plants was sampled from each plot, soil loosely attached to the roots was removed and samples were placed in plastic bags in ice and transported to the laboratory where the rhizosphere soils were collected and pooled into a single composite sample. Hence, 54 rhizosphere soil samples (3 fertilizer treatments x 6 sampling time-points x 3 replications) were obtained for microbiome analysis.

### Soil chemical analysis

Soil properties were determined as in Mofini et al. [51]. Briefly, soil pH was determined with a soil-to-water ratio of 1:2.5. Soil nitrate (NO_3_^−^) and ammonium (NH_4_^+^) were extracted with 2 M KCl and were quantified by Bran + Luebbe GmbH AutoAnalyzer III. Total carbon (C) and total nitrogen (N) contents were quantified using Elemental Analyzer (Flash EA 1112 series, ThermoFinnigan, France). Soil available phosphorus (AP) was extracted using sodium bicarbonate and then measured by the molybdenum-blue method. The P concentration was determined after dry mineralization by inductively coupled plasma atomic emission spectrometry (ICP-AES). Electrical conductivity (EC) and salinity were measured with a digital conductivity meter.

### DNA extraction, PCR amplification and sequencing

DNA was extracted from 250 mg of each rhizosphere soil sample using the FastDNA Spin Kit for Soil (MP Biomedicals, Fountain Parkway, Solon, OH, USA), according to the manufacturer’s instructions. DNA concentration and purity were determined using a Nanodrop ND-2000 UV-VIS spectrophotometer (NanoDrop Technologies, Wilmington) and DNA samples were stored at -20 °C.

Amplification and sequencing of bacterial and archaeal DNA were performed by MR DNA (www.mrdnalab.com, Shallowater, TX, USA) by targeting the V4 hypervariable region of the 16 S rRNA gene. Bacterial DNA was amplified by using the universal primers 515 F/806R [52], while archaeal DNA was amplified with the primers 349 F/806R [53]. After amplification, the quality and relative concentration of the amplicons were checked by migration on 2% agarose gel. Multiple replicates were pooled together in equal proportions based on their molecular weight and DNA concentrations. Pooled DNA samples were purified using calibrated Ampure XP beads and then the amplicons were used to prepare DNA libraries following Illumina Truseq DNA library preparation protocol. Sequencing was performed on a MiSeq Illumina platform (2 × 300) following the manufacturer’s guidelines.

### Sequence analysis

Sequence data were processed using MR DNA analysis pipeline (www.mrdnalab.com, Shallowater, TX, USA) as described in Mofini et al. [51]. In summary, raw Illumina MiSeq paired-end reads were assembled, and sequences were demultiplexed and formatted for processing using a Python script (http://drive5.com/usearch/manual/uparse_pipeline.html). Sequences were then separately quality-filtered and clustered into operational taxonomic units (OTUs) using the UPARSE algorithm [54]. Briefly, sequences were quality-filtered allowing a maximum *e*-value of 0.5. Reads were then trimmed to 240-bp length, dereplicated and sorted by abundance, and singletons were removed prior OTU determination at 97% sequence similarity threshold. Then, chimeric sequences were screened and removed using UCHIME [55] against the Gold database [56]. Finally, bacterial and archaeal OTU tables were generated by mapping reads back to the representative prokaryotic databases. The taxonomic affiliation of each OTU was obtained using BLASTn against a curated database derived from GreenGenes [57], RDPII (http://rdp.cme.msu.edu), and NCBI (www.ncbi.nlm.nih.gov). The generated raw sequence data were deposited in figshare (10.6084/m9.figshare.20348949).

### Alpha diversity

Prior alpha diversity analysis, the OTU tables of bacteria and archaea were rarefied to 14,824 and 23,889 sequences per sample respectively, using the rarefy_even_depth function in the phyloseq package [58]. Alpha diversity was then estimated by the Shannon index at each rarefaction level for bacteria and archaea. After checking for normality (Shapiro–Wilk test) and homoscedasticity (Levene test), a linear mixed-effects (LME) model fit by restricted maximum likelihood (REML) was used to test the effect of long-term fertilization and developmental stage on alpha diversity, using the lmer function in the lme4 package [59]. The LME model was:

Shannon index ~ Fertilization x Developmental stage/sampling time-point + (1|Block).

Where x indicates an interaction term, sampling time-point nested within developmental stage and plot block included as random factor to control for spatial variation.

The significance of fixed effects was assessed by the type II tests using the Anova function in the car package [60]. Whenever Anova revealed a significant effect of a given fixed factor, pairwise comparisons were conducted using the emmeans package with Tukey’s adjusted *p*-values [61]. The variance explained by the fixed factors (marginal R^2^) and those explained by both the fixed and random factors (conditional R^2^) were calculated with the r.squaredGLMM function in the MuMIn package [62].

### Beta diversity

Prior to beta diversity analysis, the OTU sequence counts of bacteria and those of archaea were normalized using the Trimmed Means of M-values (TMM) with the edgeR package [63] and the normalized counts were expressed as relative abundance counts per million (CPM). The major variance components of beta diversity in bacteria and archaea were determined by performing unconstrained principal coordinate analysis (PCoA) based on Bray-Curtis dissimilarities using the phyloseq package [58]. The effects of long-term inorganic fertilization and developmental stage on community dissimilarity were then tracked by permutational multivariate analysis of variance (PERMANOVA) using the adonis2 function in the vegan package [64]. The PERMANOVA model was run with 9999 permutations constrained within block and sampling time-point nested within developmental stage. The Mantel test based on Spearman’s correlations was performed to explore the relationship between microbial communities and soil properties using the vegan package [36].

Dataset of each kingdom (bacteria and archaea) were then separated into three subsets according to developmental stage (tillering, panicle initiation and booting) for in-depth analyses. For each data subset, we only kept OTUs that had at least three sequences and were found in at least three samples. The OTU sequence counts were subsequently TMM-normalized and the normalized counts were expressed as relative abundance CPM. We then performed canonical analysis of principal coordinates (CAP) to determine the effects of long-term inorganic fertilization and sampling time-point on each microbial community at each developmental stage using the phyloseq package [58]. Whenever significant effect was detected with PERMANOVA, pairwise comparisons were conducted using the pairwise.adonis function in the pairwiseAdonis package [65]. The analysis of multivariate homogeneity of group dispersions (BETADISP) was performed using the betadisper function, and significance was assessed using a permutation test with the permutest function in the vegan package [64].

### Long-term inorganic fertilization sensitive taxa

For each kingdom (bacteria and archaea), we used complementary approaches to identify the OTUs responsible for the observed effects of long-term inorganic fertilization at each developmental stage [66]. Correlation-based indicator species analysis was performed using the multipatt function in the indicspecies package [67] to calculate the point-biserial correlation coefficient (r) of an OTU’s positive association to one or a combination of long-term inorganic fertilizations. Likelihood ratio test (LRT) in the edgeR package [63] was then used to test for differential OTU abundance between one or more of the long-term inorganic fertilizations. Thus, OTUs whose abundances differed between one or more of the long-term inorganic fertilizations at a false discovery rate (FDR) corrected value of *p* < 0.05 were considered to be long-term inorganic fertilization responsive. Thereafter, the OTUs confirmed by both indicator species analysis and LRT were defined as long-term inorganic fertilization sensitive (*lifs*) OTUs. For each kingdom, an LME model was used to test for differences in relative abundance of *lifs* OTUs between developmental stages.

The enrichment/depletion patterns of bacterial and archaeal *lifs* OTUs at each developmental stage were visualized by drawing ternary plots using the ggtern package [68]. The same approach was conducted to identify *lifs* taxa at the higher taxonomic levels (phylum for bacteria, class for archaea because they contain a small number of phyla, and family for both kingdoms). The Proteobacteria phylum was also divided into its respective classes because it accounted for a large proportion of the bacterial community in our data set.

### Microbial inter-kingdom co-occurrence networks

The core bacterial and archaeal microbiomes on a 75% prevalence threshold were characterized using the microbiome package [69] to identify highly prevalent taxa that are present in the majority of samples at each developmental stage [51]. The core bacterial and archaeal microbiomes were combined, and microbial inter-kingdom co-occurrence networks were constructed to infer intra- and inter-kingdom interactions of OTUs at each developmental stage. For this, Spearman’s correlation between all pairs of bacterial and archaeal OTUs was performed based on their TMM-normalized CPM counts. We then considered only correlations with r > 0.6 and those with r < -0.6 associated with FDR-adjusted *p*-values < 0.01 [51]. The Gephi software (v0.9.2; https://gephi.org) was used to visualize the microbial inter-kingdom networks and estimate node-level topological features (degree, betweenness centrality, closeness centrality and eigenvector centrality) and network-level topological features (average degree, average path length, network diameter, graph density, modularity and clustering coefficient) for each developmental stage [51, 70]. For each microbial inter-kingdom network, nodes correspond to bacterial or archaeal OTUs, and edges correspond to strong correlations inferred from their TMM-normalized CPM counts. The OTUs belonging to the top 2% of degree and betweenness centrality were identified as potential hub OTUs [51, 71]. Unless mentioned otherwise, all statistical analyses were conducted in R v4.1.3 and all statistical tests were considered significant at *p* < 0.05.

## Results

### General traits of the rhizosphere bacterial and archaeal community of field-grown rice

**Fig. 1:**
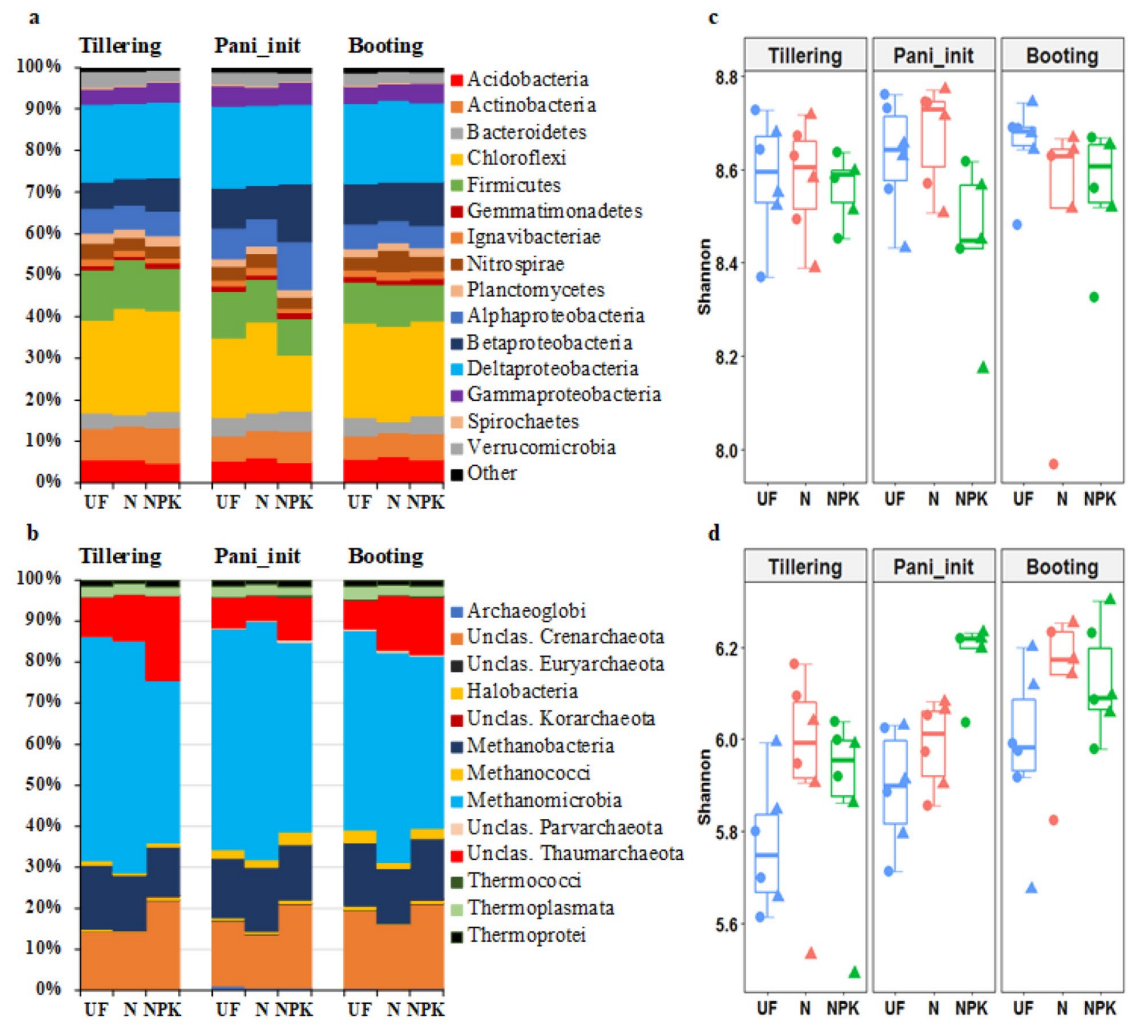
Relative OTU abundances (counts per million, CPM) and Shannon index of bacteria (a and c) and archaea (b and d) inhabiting the rice rhizosphere at tillering, panicle initiation (Pani_init) and booting stage in unfertilized (UF), N-fertilized (N) and NPK-fertilized (NPK) plots. The relative OTU abundances of bacteria are given at phylum level (class level for *Proteobacteria*) and those of archaea at class level (including both well classified and unclassified (Unclas.)). For Shannon index, circles and triangles represent the first (1-day prior fertilization) and second time-point sampling (two days post fertilization), respectively

A total of 31 bacterial phyla including 66 classes were found across all soil samples. The most dominant bacterial phyla (classes for *Proteobacteria*) in terms of relative abundance were *Chloroflexi* (13.34–25.62%), *Deltaproteobacteria* (18.21–19.72%) and *Firmicutes* (8.67–12.01%), which together accounted for 51.23% of the bacterial sequences (Fig. 1a). For archaea, we recorded 5 phyla and 13 classes across all soil samples. *Methanomicrobia* (39.54–58.19%) was the most dominant class, followed by unclassified *Crenarchaeota* (12.89–21.85%) and *Methanobacteria* (12.05–15.69%) (Fig. 1b).

Alpha diversity (Shannon index) of the bacterial community was greater than those of the archaeal community across all samples (Fig. 1c-d). For bacteria, we detected a significant interaction between fertilization and developmental stage on Shannon index (*Chi*^2^ = 12.71, *df* = 4, *p* = 0.013, Additional file 1: Table S1). However, compared to the unfertilized treatment, the long-term application of N and NPK-fertilizer did not significantly affect the bacterial alpha diversity at any of the three developmental stages (Fig. 1c). For archaea, we detected significant effects of fertilization (*Chi*^2^ = 14.77, *df* = 2, *p* = 0.001) and developmental stage (*Chi*^2^ = 20.28, *df* = 2, *p* < 0.001) on the Shannon index, while there was no significant interaction (Additional file 1: Table S1). This indicates that the inorganic fertilization and the developmental stage independently affected alpha diversity in archaeal communities. We observed a trend in which archaeal alpha diversity increased in response to N and NPK-fertilization and from tillering to booting stage (Fig. 1d).

For both bacteria and archaea, the NPK-fertilized soil was separated from the N-fertilized and unfertilized soil along the first PCoA axis (Additional file 2: Fig S1a-b) and this observation was confirmed by PERMANOVA (*F* = 3.760, *R*^2^ = 0.133, *p* < 0.001 for bacteria and *F* = 7.039, *R*^2^ = 0.239, *p* < 0.001 for archaea; Additional file 1: Table S2). Moreover, significant increases in soil pH, total P and assimilable P were observed in the order unfertilized < N-fertilized < NPK-fertilized soil, while Mantel’s test showed significant correlations between soil properties especially soil pH, NH_4_^+^-N, total C, total N and total P for both bacterial and archaeal community (Additional file 1: Table S3). We further observed a microbial distribution corresponding to the plant developmental stage, especially bacteria across the axis 3 of the PCoA plot (Additional file 2: Fig. S1c-d). PERMANOVA again revealed that the effect of developmental stage was significant for both bacteria (*F* = 2.293, *R*^2^ = 0.081, *p* < 0.001; Additional file 1: Table S2) and archaea (*F* = 1.371, *R*^2^ = 0.047, *p* = 0.023; Additional file 1: Table S2).

### Long-term fertilization effects on microbial communities inhabiting the rice rhizosphere at different developmental stages

**Fig. 2:**
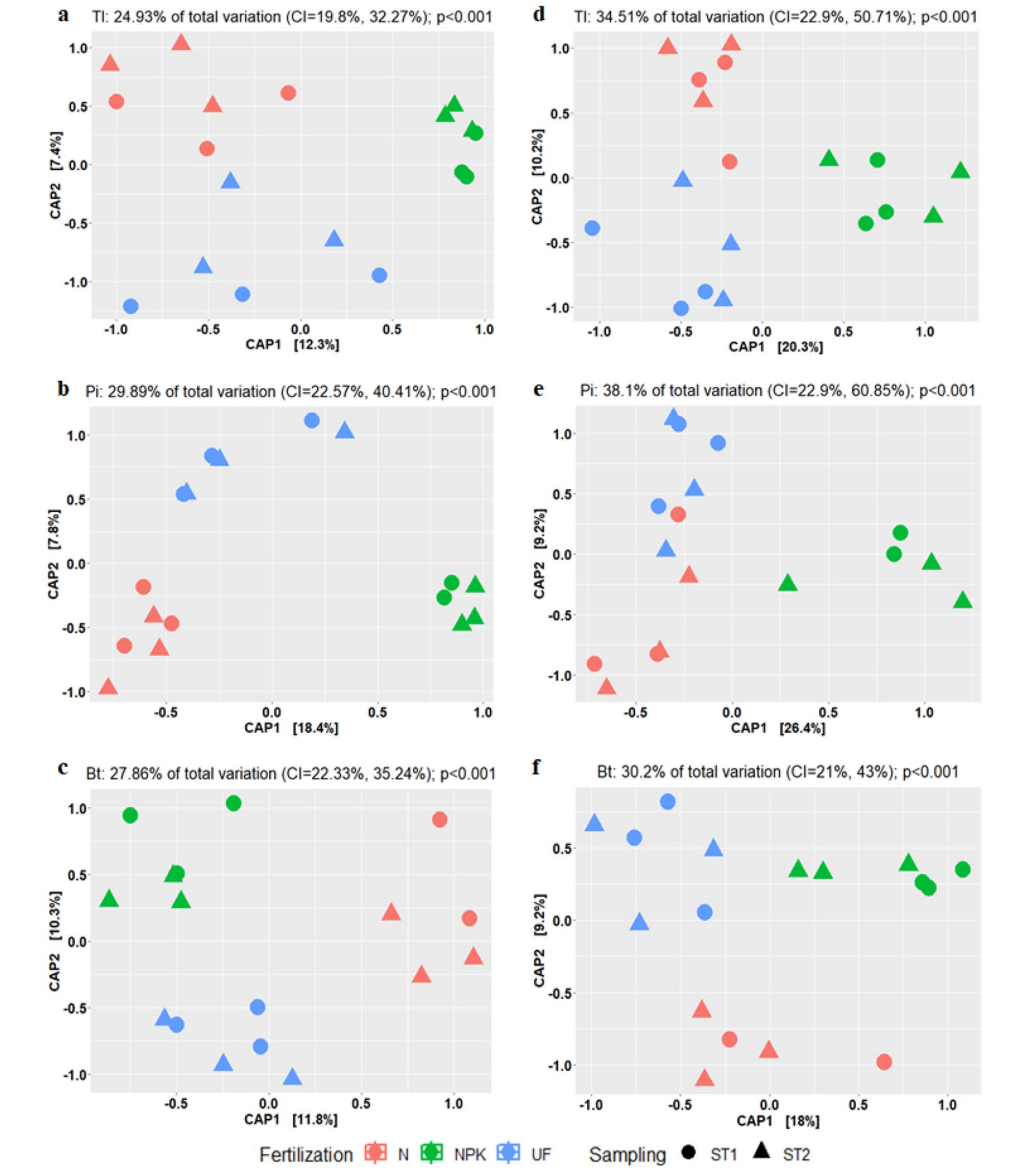
CAP ordinations depicting the effects of long-term fertilization (NPK-fertilization (NPK), N-fertilization (N), and non-fertilization control (UF)) and sampling time-point (ST1 and ST2) on bacterial (a, b and c) and archaeal (d, e and f) communities inhabiting the rice rhizosphere at tillering (Tl, a and d), panicle initiation (Pi, b and e) and booting stage (Bt, c and f). The explained fraction of the total variance (with 95% confidence interval, significance assessed with 9999 permutations) are given above the plots. For each developmental stage, ST1 ST2 refer to the first and second sampling time-point, respectively

The partial CAP, constrained by both long-term fertilization and sampling time-point, showed significant effects of long-term fertilization on both bacterial and archaeal community at tillering, panicle initiation and booting stage (Fig. 2).

No significant effects of sampling time (pre and post fertilization at the same developmental stage) were observed on both bacterial and archaeal communities at different developmental stages. This observation was confirmed by PERMANOVA (Additional file 1: Table S4). The pairwise comparisons for bacterial composition revealed that, at tillering stage, unfertilized and N-fertilized rhizosphere soil harbored not significantly different communities, while they differed significantly from those in the NPK-fertilized rhizosphere soil. In contrast, the three treatments (unfertilized, N-fertilized and NPK-fertilized) harbored dissimilar bacterial communities both at panicle initiation and at booting stage (Additional file 1: Table S4). For archaeal communities, significant differences were observed between unfertilized, N-fertilized and NPK-fertilized soil at tillering and booting stages. Whereas, at panicle initiation, just the NPK-fertilized was significant different compared with the unfertilized and the N-fertilized rhizosphere soils (Additional file 1: Table S4). Furthermore, the Betadisper analysis revealed significant differences in group dispersion for the archaeal community at tillering (*F* = 4.024, *df* = 2, *p* = 0.028) and panicle initiation stages (*F* = 3.237, *df* = 2, *p* = 0.009). Conversely, no significant differences in group dispersion were observed for bacterial community at different developmental stages, suggesting that the differences between fertilization regimes were mainly driven by true biological differences (Additional file 1: Table S4). Overall, our results indicate that the effect of long-term fertilization affect the compositions of the rhizosphere microbial communities depending on the developmental stage, and that the bacterial and archaeal community differed in their response to long-term N and NPK-fertilization.

### Distribution of long-term inorganic fertilization sensitive taxa across developmental stages of field-grown rice

**Fig. 3:**
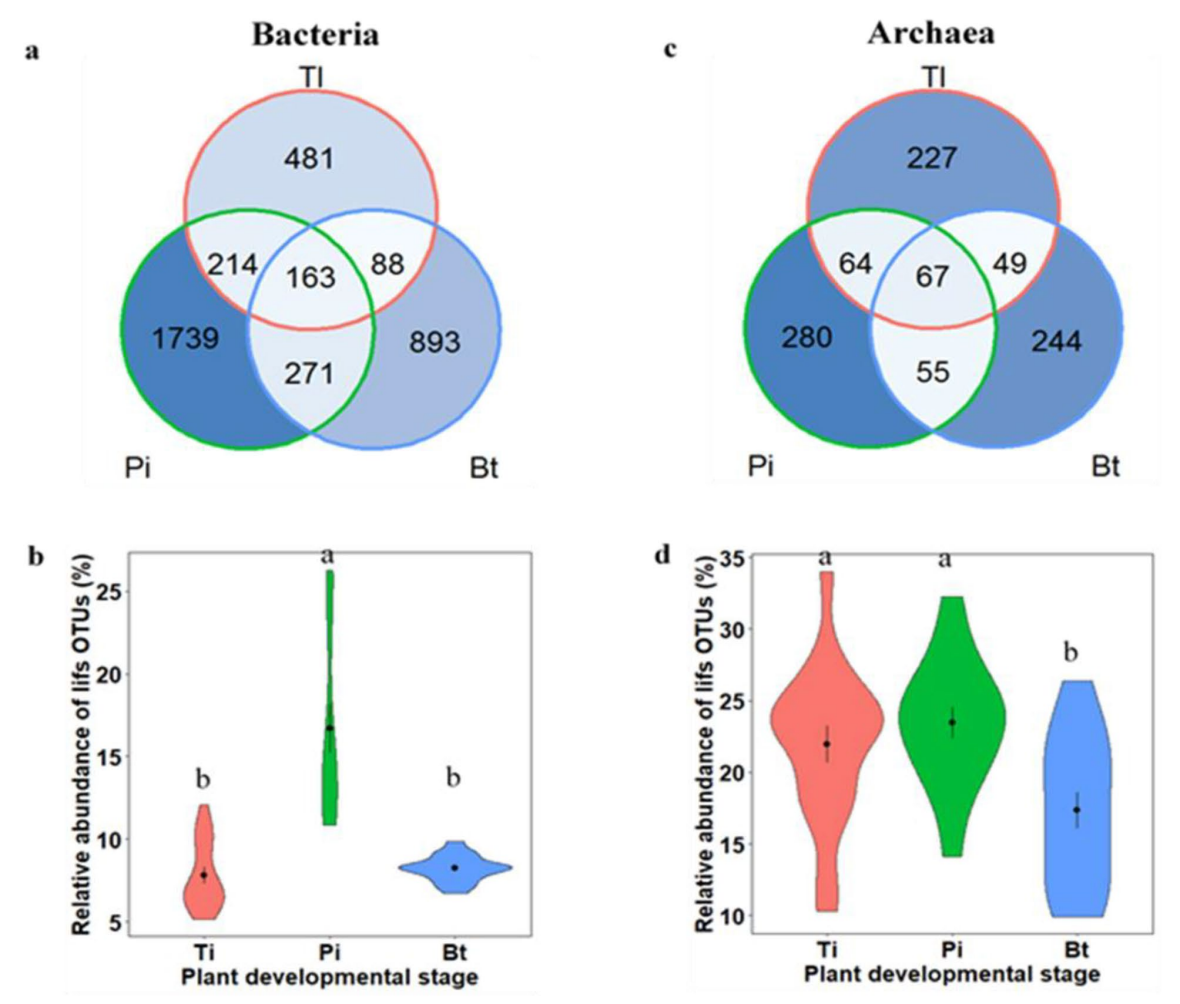
Number and relative abundance of long-term inorganic fertilization sensitive (lifs) OTUs of bacteria (a and b) and archaea (c and d) inhabiting the rice rhizosphere at tillering (Tl), panicle initiation (Pi) and booting stage (Bt). Black dots and error bars within violins represent means and standard errors

We combined indicator species analysis and likelihood ratio test (LRT) to determine the long-term inorganic fertilization sensitive (*lifs*) OTUs (Additional file 2: Fig. S2). We identified 3849 and 986 *lifs* OTUs of bacteria and archaea respectively, across the different developmental stages (Fig. 3a-b). The bacterial community included 946, 2387 and 1415 *lifs* OTUs contributing to 7.81, 16.67 and 8.22% of the total abundance at tillering, panicle initiation and booting stage, respectively (Fig. 3a-c). For archaea, we found 407, 466 and 415 *lifs* OTUs accounting for 22, 23.40 and 17.30% of the total abundance at tillering, panicle initiation and booting stage, respectively (Fig. 3b-d).

**Fig. 4:**
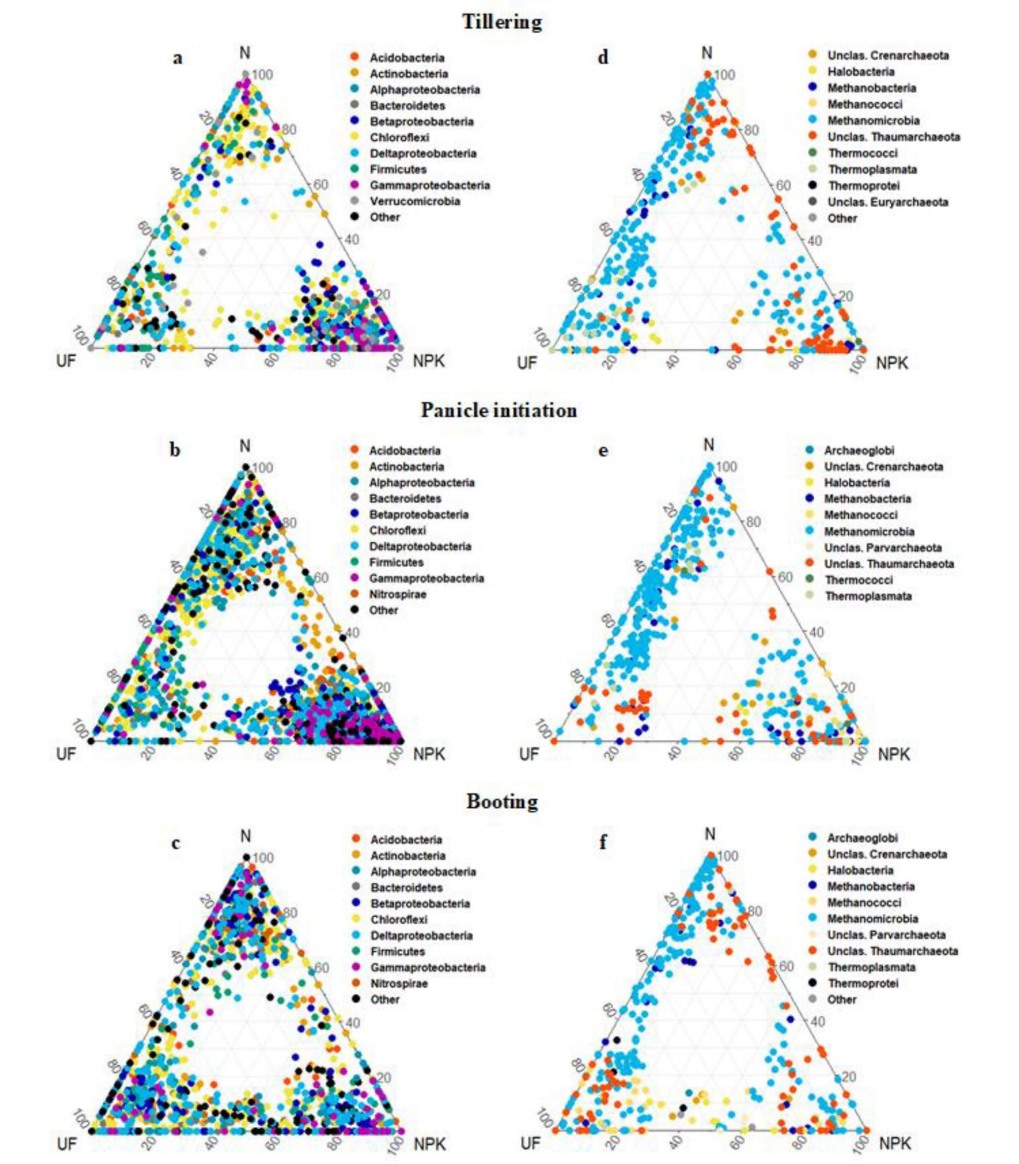
Ternary plots depicting the enrichment/depletion patterns of long-term inorganic fertilization sensitive (*lifs*) OTUs of rhizosphere bacteria (a, b and c) and archaea (d, e and f) in unfertilized (UF), N-fertilized (N) and NPK-fertilized soil (NPK) at tillering, panicle initiation and booting stage of field-grown rice. The ternary plots were constructed based on the mean relative abundances (counts per million, CPM) of *lifs* OTUs. The bacterial and archaeal *lifs* OTUs are colored based on the phyla (classes for *Proteobacteria*) and classes (well classified and unclassified (Unclas.)) to which they belong

A statistically significant effect of developmental stage on the relative abundance of *lifs* OTUs was detected for both kingdoms, with a larger magnitude for bacteria (*Chi*^2^ = 62.73, *df* = 2, *p* < 0.001) compared to archaea (*Chi*^2^ = 13.42, *df* = 2, *p* = 0.001). In addition, dynamic enrichment/depletion patterns of bacterial and archaeal *lifs* OTUs in unfertilized, N-fertilized and NPK-fertilized soil were observed across developmental stages (Fig. 4). For instance, the bacterial *lifs* OTUs were preferentially enriched in NPK-fertilized soil, followed by N-fertilized soil at panicle initiation, whereas they were preferentially depleted in NPK-fertilized soil at booting stage (Fig. 4a-c). For archaea, the *lifs* OTUs were also preferentially depleted in NPK-fertilized soil as compared to unfertilized and N-fertilized soil at tillering and booting stage (Fig. 4d-f).

On the other hand, 7 bacterial phyla and 6 archaeal classes were identified as sensitive to long-term inorganic fertilization across the different developmental stages (Additional file 2: Fig. S3). At tillering stage, *Epsilonproteobacteria* was significantly depleted, whereas unclassified *Parvarchaeota* was enriched in NPK-fertilized. At panicle initiation, *Alphaproteobacteria* and *Betaproteobacteria* were significantly enriched, whereas *Chlorobi*, *Chloroflexi*, *Ignavibacteriae*, *Verrucomicrobia*, *Methanomicrobia* and *Thermoplasmata* were depleted in NPK-fertilized soil. At booting stage, unclassified *Thaumarchaeota* were enriched, whereas *Halobacteria* and *Methanococci* were depleted in N-fertilized soil (Additional file 2: Fig. S3). Furthermore, 57 and 3 bacterial and archaeal families, respectively, were identified as sensitive to long-term inorganic fertilization across the different developmental stages (Additional file 2: Fig. S4). Although some of those families exhibited their sensitivity to long-term inorganic fertilization either at one, two or three developmental stages, most of them were preferentially sensitive at panicle initiation (Additional file 2: Fig. S4).

Taken together, the effect of rice developmental stage on microbial sensitivity to long-term inorganic fertilization was more pronounced for bacterial than for archaeal communities. Within the bacterial communities, a higher relative abundance of *lifs* taxa at panicle initiation compared to at tillering and booting stages was identified.

### Microbial inter-kingdom co-occurrence patterns in the rhizosphere core microbiome across developmental stages of field-grown rice

**Fig. 5:**
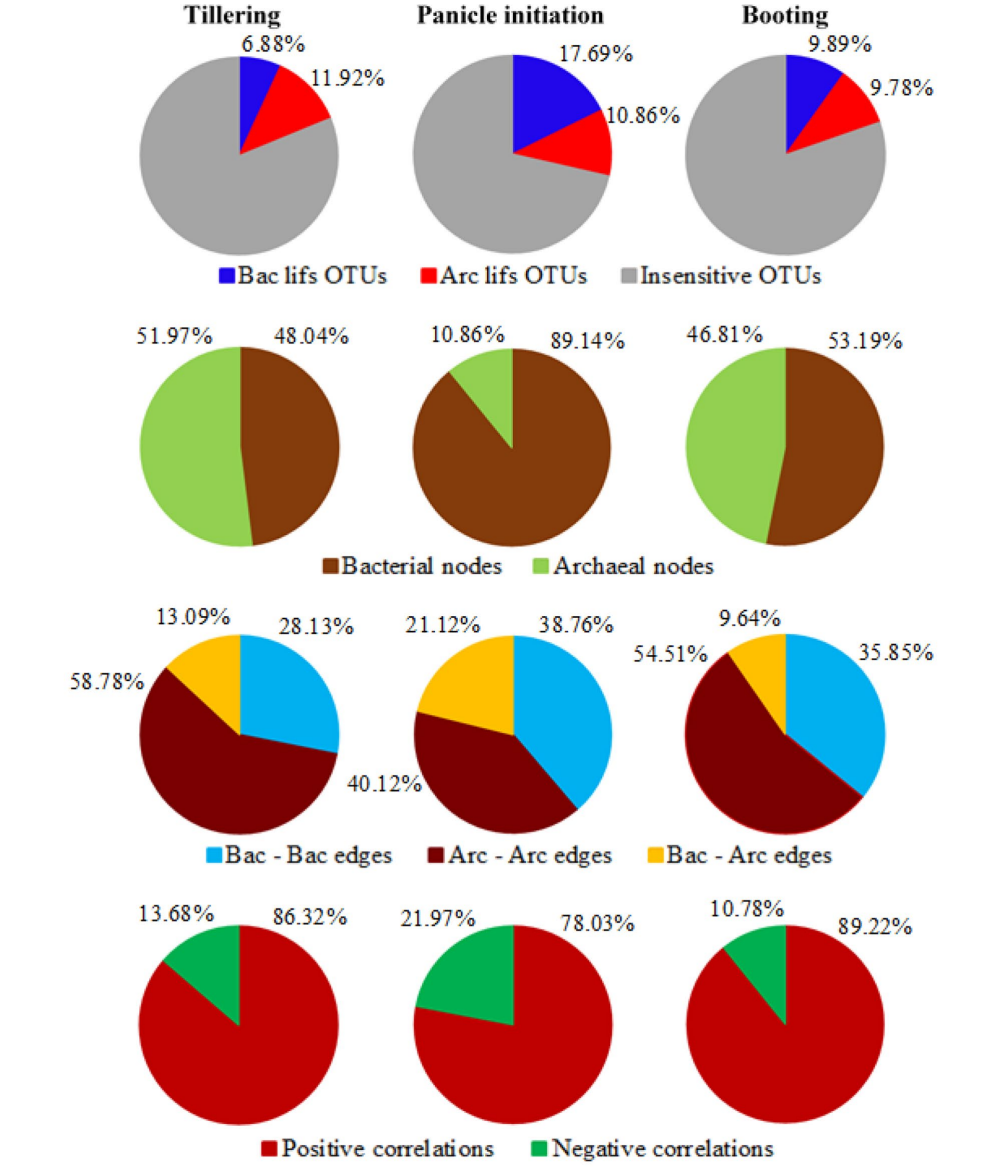
Proportions of bacterial and archaeal *lifs* OTUs, nodes, edges, and positive and negative correlations in each inter-kingdom network. Bac and Arc indicate bacteria and archaea, respectively

We analyzed the differences in the core microbiomes (treatment-independent) at the different developmental stages by investigating the taxa co-occurrence patterns using microbial inter-kingdom network analysis. The microbial inter-kingdom network obtained at tillering stage contained 814 nodes, those at panicle initiation contained 1040 nodes and those at booting stage contained 910 nodes (Additional file 1: Table S5, Additional file 2: Fig. S5). Furthermore, the microbial inter-kingdom network obtained at panicle initiation displayed a higher proportion (28.56%) of *lifs* OTUs than those obtained at booting (19.67%) and tillering stage (18.80%) (Fig. 5). The proportion of bacterial nodes increased from tillering to panicle initiation and decreased to booting stage, while those of archaeal nodes followed the opposite trend. Similarly, the proportion of bacteria – bacteria edges, bacteria – archaea edges and negative correlations increased from tillering to panicle initiation and decreased to booting stage, whereas those of archaea – archaea edges and positive correlations followed opposite trends (Fig. 5, Additional file 1: Table S5).

In addition, 17 potential hub OTUs were identified across the three developmental stages (Additional file 1: Table S6). Of those potential hub OTUs, 2 belonging to archaea (*Methanobacterium* and *Methanosaeta*) were identified at tillering stage, 9 including 2 bacteria (*Gemmatimonas* and *Pirellula*) and 7 archaea (four *Methanobacterium*, two *Methanosaeta* and one *Methanosphaerula*) at panicle initiation, and 6 belonging to archaea (five *Methanosaeta* and one *Methanobacterium*) at booting stage (Additional file 1: Table S6). Among these 17 potential hub OTUs, 12 were also identified as *lifs* OTUs (Additional file 1: Table S6). Together these results reveal the dynamics of bacteria and archaea co-occurrence patterns in the rice rhizosphere, with differentiated bacterial and archaeal pivotal roles in the microbial inter-kingdom networks across developmental stages.

## Discussion

Understanding how the plant host (e.g., genotype, developmental stage), environmental factors (e.g., edaphic properties, climate) and agricultural management (e.g., tilling, fertilization) modulate the soil microbial communities is essential to develop sustainable strategies for efficiently harness the soil microbiome to increase crop production and soil resilience [72–74]. Here, we investigated the effect of long-term N and NPK-fertilization on bacterial and archaeal communities inhabiting the rice rhizosphere at three developmental stages (tillering, panicle initiation and booting) in the Senegal River Delta.

First, we found that the bacterial community was dominated by *Proteobacteria*, *Chloroflexi*, *Firmicutes*, *Actinobacteria*, and *Acidobacteria* across treatments. For the archaeal community, unclassified *Crenarchaeota*, *Methanomicrobia*, *Methanobacteria* and unclassified *Thaumarchaeota* represented the most abundant classes in the rice rhizosphere. These results are consistent with previous studies from bacterial and archaeal kingdoms in rice paddy rhizospheres of different environments [75–77].

We next analyzed the impact of long-term inorganic fertilization and developmental stage on microbial alpha diversity in the rice rhizosphere. We observed that the alpha diversity was greater for the bacterial than archaeal community, irrespective of fertilization regime and developmental stage. In addition, we found that the interaction between inorganic fertilization and developmental stage had a limited impact on bacterial alpha diversity. This observation is in accordance with previous reports showing that environmental factors (i.e., soil nutrients or agricultural management systems) as well as developmental stage have a limited effect on bacterial alpha diversity [78, 79].

Looking further into microbial community structure, we observed that long-term inorganic fertilization resulted in significant shifts in both bacterial and archaeal community structure, and that the N and NPK-fertilization differentially affected them. Soil pH, NH_4_^+^-N, total C, total N and total P were identified as key edaphic factors shaping the rice rhizosphere bacterial and archaeal communities. Rice developmental stage was also found to be a significant factor explaining the observed variation in rhizosphere bacterial and archaeal community structures in accordance with previous observations [35, 36, 80]. The changes in root exudation during rice growth might be the cause of the observed effect of developmental stage on rhizosphere microbial communities [39, 40, 81]. Indeed, root exudation rate increases from seedling to flowering stage and decreases to maturity, meanwhile the exudation of sugars was substituted by those of organic acids with advancing rice plant growth [82].

We next identified 3849 and 986 long-term inorganic fertilization sensitive (*lifs)* OTUs for bacteria and archaea respectively, across the different developmental stages. These OTUs have the potential to function as indicator taxa [66]. Moreover, our results indicate that the microbial communities inhabiting the rice rhizosphere at panicle initiation are more sensitive to long-term inorganic fertilization than those at tillering and booting stages. Indeed, the highest *lifs* OTUs relative abundances for both bacterial and archaeal communities were obtained at panicle initiation. This long-term fertilization sensitivity pattern of microbial communities across developmental stages may be correlated to the rhizosphere microbial activity. In fact, the rice rhizosphere enzymatic activity as proxy of microbial activity was shown to increase up to panicle initiation and decline thereafter until maturity [83, 84]. In other words, the activities of dehydrogenase, urease, acid phosphatase and alkaline phosphatase increased in rhizosphere soil from tillering to panicle initiation and decreased to maturity of rice grown under different crop management conditions [83]. Furthermore, it was shown that the panicle initiation is among the most sensitive developmental stage to biotic and abiotic stresses in rice [85–87].

The differential microbial sensitivity to long-term inorganic fertilization across developmental stages supports the idea that rice may adjust its root metabolism and exudation to recruit specific rhizosphere microbial taxa in order to meet the changing demand for nutrients and/or regulate immune responses [88, 89]. Our results also show that the long-term inorganic fertilization impact across developmental stages was more profound on the bacterial than the archaeal communities. Divergent evolutionary paths between bacteria and archaea, in relation to the availability and/or demand for resources and edaphic factors might explain this observation. Congruently, Valentine [90] suggested that archaea have evolved to thrive with energy stress, while bacteria can adapt to maximize the availability of energy and other resources.

In addition, dynamic enrichment/depletion patterns of *lifs* OTUs in unfertilized, N-fertilized and NPK-fertilized soil were observed across developmental stages, suggesting the existence of distinctly different ecological niches suitable for either copiotrophic or oligotrophic microbial taxa [91]. Partial support for this hypothesis comes from the observation that phyla/classes such as *Alphaproteobacteria* and *Betaproteobacteria*, whose members are often described to have a copiotrophic lifestyle, [11, 92] were preferentially enriched in NPK-fertilized soil. Whereas *Chloroflexi*, whose members exhibit an oligotrophic lifestyle, [93, 94] were preferentially depleted in NPK-fertilized soil at panicle initiation. *Thaumarchaeota*, which also includes copiotrophic taxa [95, 96], were preferentially enriched in N and NPK-fertilized soil at booting stage.

On the other hand, the analysis of taxa co-occurrence patterns from core microbiomes of the rice rhizosphere revealed that the inter-kingdom network obtained at panicle initiation displayed a higher proportion of *lifs* OTUs than those obtained at booting and tillering stages. This mirrors our findings from the analysis of the whole bacterial and archaeal community, while indicating that even if are prevalent, i.e., shared by all or most samples, certain taxa can become sensitive to long-term inorganic fertilization at a given developmental stage. Yet, the proportion of bacterial nodes increased from tillering to panicle initiation and decreased to booting stage, while that of archaeal nodes followed opposite trends. There results suggest that rice may selectively recruit and maintain core microbiome members and thereby modulate microbe-microbe and soil-microbe interactions to satisfy its abiotic and biotic requirements across developmental stages [72, 97, 98]. For instance, Xiong et al. [36] observed in maize that the bacterial and fungal proportions showed opposite shifting trends across three developmental stages. In the present study, the panicle initiation displayed a higher proportion of negative correlations than the tillering and booting stage. This may reflect either more competition between microbial taxa for limiting resources, targeted allelopathy or distinctive environmental niches at panicle initiation than at other developmental stages [99, 100].

Furthermore, the microbial inter-kingdom networks obtained at tillering, panicle initiation and booting stage displayed dissimilar groups of potential hub taxa, suggesting that rice develops distinct dialogues with its microbiome across developmental stages. These potential hub taxa may play crucial roles in the assembly of microbial communities at each developmental stage [101]. Of the 17 potential hub taxa, 15 fell into three genera (*Methanosaeta*, *Methanobacterium*, and *Methanosphaerula*) belonging to three methanoarchaeal orders (Methanosarcinales, Methanobacteriales, and Methanomicrobiales), that share the ability to produce methane [102, 103]. Notably, methanogenic archaea have been identified as hub or keystone taxa in various ecological habitats such as the wheat rhizosphere [104], permafrost [105], human gastrointestinal tract [106], and in holobionts [107]. The high prevalence of methanogenic archaea as hub taxa may be linked to their ability to convert diverse bacterial end products of fermentation such as carbon dioxide, hydrogen, methanol, and acetate (in case of Methanosarcinales), making them highly flexible in forming syntrophic relationship with a broad range of bacteria [108–110]. In addition, methanogenic archaea can fix nitrogen [18, 111, 112] and make it available for methane-consuming microbial consortia [113]. Through these syntrophic interactions with other microbial communities, the methanogenic archaea may significantly contribute to carbon cycling in paddy fields [114]. Besides methanogenic archaea, two bacteria, *Gemmatimonas* and *Pirellula*, were identified as potential hub taxa at panicle initiation. *Gemmatimonas* is known to play an important role in soil organic carbon dynamics by using metabolic products of cellulose such as acetate and propionate as the sole carbon source [115]. The contribution of *Gemmatimonas* to carbon, nitrogen and phosphorus transformation through the decomposition of organic matter in the soil [116, 117], combined to its ability to fix nitrogen [118, 119] and potential role in plant disease suppression [120, 121], may underpin its pivotal role as keystone in various environments [104, 122, 123]. *Pirellula* is involved in nutrient dynamics in soil through nitrogen cycling [124]. Indeed, *Pirellula* can generate N_2_ by using NO_2_^−^-N obtained from NO_3_^−^-N denitrification to oxide NH_4_^+^-N under hypoxic or anaerobic environment [125, 126]. The impact of these potential hub taxa on the fitness of rice plants remains to be elucidated. Furthermore, the use of technologies such as metatranscriptomics could help to expand our understanding of the microbial community’s functions in this West African Sahelian agroecosystem.

## Conclusions

We observed significant change in both rhizosphere bacterial and archaeal community compositions in response to long-term (27 years) N and NPK-fertilization. The bacterial and archaeal communities differed in their response to N and NPK-fertilization. The microbial communities inhabiting the rice rhizosphere at panicle initiation appear to be more sensitive to long-term inorganic fertilization than those at tillering and booting stage. However, the developmental stage impact on microbial sensitivity to long-term inorganic fertilization was more pronounced for bacterial than archaeal community. Furthermore, our results reveal dynamics of bacteria and archaea co-occurrence patterns in the rice rhizosphere, with differentiated bacterial and archaeal pivotal roles in the microbial inter-kingdom networks across developmental stages. Based on these results, we speculate that the core taxa, especially the putative hub taxa, would have significant influence on development and productivity of rice through nutrient cycling and beneficial biotic interactions such as microbe-microbe, microbe-fauna and microbe-plant interactions. By identifying one of the critical rice developmental stages during which the rhizosphere microbial communities are highly sensitive to inorganic fertilization as well as the fertilization sensitive microbial taxa, our results open new avenues for developing strategies in microbiome engineering to mitigate biotic and abiotic stress and improve rice yields. Future research should focus on these core and potential hub taxa to develop new-generation bio-fertilizers for more resilient and sustainable agriculture.

## Electronic supplementary material

Below is the link to the electronic supplementary material.

Supplementary Material 1

Supplementary Material 2

## Data Availability

The datasets generated during the current study are available in the figshare repository (10.6084/m9.figshare.20348949).

## Declarations

### List of Abbreviations

All abbreviations are defined in the text.

## References

[1] Van Ittersum MK, Van Bussel LG, Wolf J, Grassini P, Van Wart J, Guilpart N, Cassman KG (2016). Can sub-saharan Africa feed itself?. Proc Natl Acad Sci.

[2] Arif I, Batool M, Schenk PM (2020). Plant microbiome engineering: expected benefits for improved crop growth and resilience. Trends Biotechnol Review.

[3] Ding LJ, An XL, Li S, Zhang GL, Zhu YG (2014). Nitrogen loss through anaerobic ammonium oxidation coupled to iron reduction from paddy soils in a chronosequence. Environ Sci Technol.

[4] Ge T, Li B, Zhu Z, Hu Y, Yuan H, Dorodnikov M, Jones DL, Wu J, Kuzyakov Y (2017). Rice rhizodeposition and its utilization by microbial groups depends on N fertilization. Biol Fertil Soils.

[5] Yuan S, Linquist BA, Wilson LT, Cassman KG, Stuart AM, Pede V, Miro B, Saito K, Agustiani N, Aristya VE, Krisnadi LY (2021). Sustainable intensification for a larger global rice bowl. Nat commun.

[6] FAO. Food Outlook - Biannual Report on Global Food Markets. Rome; 2019. Licence: CC BY-NC-SA 3.0 IGO.

[7] FAO. World Food and Agriculture - Statistical Yearbook. Rome; 2021. 10.4060/cb4477en.

[8] Khush GS (2005). What it will take to feed 5.0 billion rice consumers in 2030. Plant mol biol.

[9] Ahmadi N, Audebert A, Bennett MJ, Bishopp A, de Oliveira AC, Courtois B, Diedhiou AG, Diévart A, Gantet P, Ghesquière A, Guiderdoni E, Henry A, Inukai Y, Kochian L, Laplaze L, Lucas M, Luu DT, Manneh B, Mo X, Muthurajan R, Périn C, Price A, Robin S, Sentenac H, Sine B, Uga Y, Véry AA, Wissuwa M, Wu P, Xu J (2014). The roots of future rice harvests. Rice.

[10] Diedhiou AG, Mbaye FK, Mbodj D, Faye MN, Pignoly S, Ndoye I, Djaman K, Gaye S, Kane A, Laplaze L, Manneh B, Champion A (2016). Field trials reveal ecotype-specific responses to mycorrhizal inoculation in rice. PLoS ONE.

[11] Wang J, Song Y, Ma TF, Raza W, Li J, Howland JG, Huang QW, Shen QR (2017). Impacts of inorganic and organic fertilization treatments on bacterial and fungal communities in a paddy soil. Appl Soil Ecol.

[12] Yang Y, Wang P, Zeng Z (2019). Dynamics of bacterial communities in a 30-year fertilized paddy field under different organic-inorganic fertilization strategies. Agronomy.

[13] Singh B, Ryan J (2015). Managing fertilizers to enhance soil health.

[14] Pahalvi HN, Rafiya L, Rashid S, Nisar B, Kamili AN, Dar GH, Bhat RA, Mehmood MA, Hakeem KR (2021). Chemical fertilizers and their impact on soil health. Microbiota and Biofertilizers.

[15] Savci S (2012). Investigation of effect of chemical fertilizers on environment. Apcbee Procedia.

[16] Walling E, Vaneeckhaute C (2020). Greenhouse gas emissions from inorganic and organic fertilizer production and use: a review of emission factors and their variability. J Environ Manage.

[17] Finkel OM, Castrillo G, Paredes SH, González IS, Dangl JL (2017). Understanding and exploiting plant beneficial microbes. Curr Opin Plant Biol.

[18] Soumare A, Diedhiou AG, Thuita M, Hafidi M, Ouhdouch Y, Gopalakrishnan S, Kouisni L (2020). Exploiting Biological Nitrogen fixation: a Route towards a sustainable agriculture. Plants.

[19] Soumare A, Sarr D, Diedhiou AG (2022). Potassium sources, microorganisms, and plant nutrition—challenges and future research directions: a review. Pedosphere.

[20] Van der Heijden MGA, Bardgett RD, Van Straalen NM (2008). The unseen majority: soil microbes as drivers of plant diversity and productivity in terrestrial ecosystems. Ecol Lett.

[21] Berendsen RL, Pieterse CM, Bakker PA (2012). The rhizosphere microbiome and plant health. Trends in plant sci.

[22] Chaparro JM, Sheflin AM, Manter DK, Vivanco JM (2012). Manipulating the soil microbiome to increase soil health and plant fertility. Biol Fertil of Soils.

[23] Power AG (2010). Ecosystem services and agriculture: tradeoffs and synergies. Philosophical transactions of the royal society B. Biol sci.

[24] Bardgett RD, van der Putten WH (2014). Belowground biodiversity and ecosystem functioning. Nat.

[25] Wagg C, Bender SF, Widmer F, Van Der Heijden MG (2014). Soil biodiversity and soil community composition determine ecosystem multifunctionality. Proc Natl Acad Sci.

[26] Wang Q, Jiang X, Guan D, Wei D, Zhao B, Ma M, Chen S, Li L, Cao F, Li J (2018). Long-term fertilization changes bacterial diversity and bacterial communities in the maize rhizosphere of chinese mollisols. Appl Soil Ecol.

[27] Banerjee S, Walder F, Büchi L, Meyer M, Held AY, Gattinger A, Keller T, Charles R, van der Heijden MG (2019). Agricultural intensification reduces microbial network complexity and the abundance of keystone taxa in roots. The ISME J.

[28] Liu J, Shu A, Song W, Shi W, Li M, Zhang W, Li Z, Liu G, Yuan F, Zhang S, Liu Z (2021). Long-term organic fertilizer substitution increases rice yield by improving soil properties and regulating soil bacteria. Geoderma.

[29] Geisseler D, Scow KM (2014). Long-term effects of mineral fertilizers on soil microorganisms – A review. Soil Biol Biochem.

[30] Guo P, Wang C, Jia Y, Wang Q, Han G, Tian X (2011). Responses of soil microbial biomass and enzymatic activities to fertilizations of mixed inorganic and organic nitrogen at a subtropical forest in East China. Plant Soil.

[31] Francioli D, Schulz E, Lentendu G, Wubet T, Buscot F, Reitz T (2016). Mineral vs. Organic amendments: Microbial Community structure, activity and abundance of agriculturally relevant Microbes are driven by long-term fertilization strategies. Front Microbiol.

[32] Wu Y, Lu L, Wang B, Lin X, Zhu J, Cai Z, Yan X, Jia Z (2011). Long-term field fertilization significantly alters community structure of ammonia‐oxidizing bacteria rather than archaea in a paddy soil. Soil Sci Soc Am J.

[33] Lynch JM, Brimecombe MJ, De Leij FA. Rhizosphere. e LS. 2001.

[34] de la Fuente Cantó C, Simonin M, King E, Moulin L, Bennett MJ, Castrillo G, Laplaze L (2020). An extended root phenotype: the rhizosphere, its formation and impacts on plant fitness. The Plant J.

[35] Chaparro JM, Badri DV, Vivanco JM (2014). Rhizosphere microbiome assemblage is affected by plant development. The ISME J.

[36] Xiong C, Singh BK, He JZ, Han YL, Li PP, Wan LH, Meng GZ, Liu SY, Wang JT, Wu CF, Ge AH (2021). Plant developmental stage drives the differentiation in ecological role of the maize microbiome. Microbiome.

[37] Oyserman BO, Flores SS, Griffioen T, Pan X, van der Wijk E, Pronk L, Lokhorst W, Nurfikari A, Paulson JN, Movassagh M, Stopnisek N (2022). Disentangling the genetic basis of rhizosphere microbiome assembly in tomato. Nat commun.

[38] Pérez-Jaramillo JE, Carrión VJ, Bosse M, Ferrão LF, De Hollander M, Garcia AA, Ramírez CA, Mendes R, Raaijmakers JM (2017). Linking rhizosphere microbiome composition of wild and domesticated Phaseolus vulgaris to genotypic and root phenotypic traits. ISME J.

[39] Sasse J, Martinoia E, Northen T (2018). Feed your friends: do plant exudates shape the root microbiome?. Trends Plant Sci.

[40] Pascale A, Proietti S, Pantelides IS, Stringlis IA (2020). Modulation of the root microbiome by plant molecules: the basis for targeted disease suppression and plant growth promotion. Front Plant Sci.

[41] Ibrahim A, Saito K (2022). Assessing genetic and agronomic gains in rice yield in sub-saharan Africa: a meta-analysis. Field Crops Res.

[42] Haefele SM, Thomas CL, Saito K (2022). Long-term fertility experiments for irrigated rice in the West African Sahel: Effect on macro-and micronutrient concentrations in plant and soil. Field Crops Res.

[43] Edwards JA, Santos-Medellin CM, Liechty ZS, Nguyen B, Eason S, Philips G, Sundaresan V (2018). Compositional shifts in root-associated bacterial and archaeal microbiota track the plant life cycle field-grown rice. PLoS Biol.

[44] Kumar U, Nayak AK, Shahid M, Gupta VV, Panneerselvam P, Mohanty S, Kaviraj M, Kumar A, Chatterjee D, Lal B, Gautam P (2018). Continuous application of inorganic and organic fertilizers over 47 years in paddy soil alters the bacterial community structure and its influence on rice production. Agric Ecosyst Environ.

[45] Dai X, Song D, Guo Q, Zhou W, Liu G, Ma R, Liang G, He P, Sun G, Yuan F, Liu Z (2021). Predicting the influence of fertilization regimes on potential N fixation through their effect on free-living diazotrophic community structure in double rice cropping systems. Soil Biol Biochem.

[46] McQuilken MP, Halmer P, Rhodes DJ, Burges H (1998). Application of microorganisms to seeds. Formulation of Microbial Biopesticides.

[47] Bashan Y, de-Bashan LE, Prabhu SR, Hernandez JP (2014). Advances in plant growth-promoting bacterial inoculant technology: formulations and practical perspectives (1998–2013). Plant Soil.

[48] Bado BV, Aw A, Ndiaye M (2011). Long-term effect of continuous cropping of irrigated rice on soil and yield trends in the Sahel of West Africa. Innovations as key to the Green Revolution in Africa.

[49] Djaman K, Mel VC, Ametonou FY, Namaky RE, Diallo MD, Koudahe K (2018). Effect of Nitrogen Fertilizer dose and application timing on yield and Nitrogen Use Efficiency of Irrigated Hybrid Rice under Semi-Arid Conditions. J Agri Sci Food Res.

[50] Haefele SM, Wopereis MCS, Wiechmann H (2002). Long-term fertility experiments for irrigated rice in the west african sahel: agronomic results. Field Crops Res.

[51] Mofini MT, Diedhiou AG, Simonin M, Dondjou DT, Pignoly S, Ndiaye C, Min D, Vigouroux Y, Laplaze L, Kane A (2022). Cultivated and wild pearl millet display contrasting patterns of abundance and co-occurrence in their root mycobiome. Sci Rep.

[52] Caporaso JG, Lauber CL, Walters WA, Berg-Lyons D, Huntley J, Fierer N, Owens SM, Betley J, Fraser L, Bauer M, Gormley N, Gilbert JA, Smith G, Knight R (2012). Ultra-high-throughput microbial community analysis on the Illumina HiSeq and MiSeq platforms. ISME J.

[53] Takai K, Horikoshi K (2000). Rapid detection and quantification of members of the archaeal community by quantitative PCR using fluorogenic probes. Appl Environ Microbiol.

[54] Edgar RC (2013). UPARSE: highly accurate OTU sequences from microbial amplicon reads. Nat Meth.

[55] Edgar RC, Haas BJ, Clemente JC, Quince C, Knight R (2011). UCHIME improves sensitivity and speed of chimera detection. Bioinformatics.

[56] Reddy TB, Thomas AD, Stamatis D, Bertsch J, Isbandi M, Jansson J, Mallajosyula J, Pagani I, Lobos EA, Kyrpides NC (2015). The Genomes OnLine database (GOLD) v.5: a metadata management system based on a four level (meta) genome project classification. Nucleic Acids Res.

[57] DeSantis TZ, Hugenholtz P, Larsen N, Rojas M, Brodie EL, Keller K, Huber T, Dalevi D, Hu P, Andersen GL (2006). Greengenes, a chimera-checked 16S rRNA gene database and workbench compatible with ARB. Appl Environ Microbiol.

[58] McMurdie PJ, Holmes H (2013). Phyloseq: an R Package for Reproducible Interactive Analysis and Graphics of Microbiome Census Data. PLoS ONE.

[59] Bates D, Mächler M, Bolker BM, Walker SC (2015). Fitting linear mixed-effects models using lme4. J Stat Softw.

[60] Fox J, Weisberg S (2011). An R companion to Applied Regression.

[61] Lenth R, Emmeans. Estimated Marginal Means, aka Least-Squares Means. 2020. https://cran.r-project.org/package=emmeans. 95.

[62] Barton K. Multi-Model Inference. Version 1.43.17 MuMIn.pdf (r-project.org). 2020.

[63] Robinson MD, McCarthy DJ, Smyth GK (2010). edgeR: a bioconductor package for differential expression analysis of digital gene expression data. Bioinformatics.

[64] Oksanen J, Blanchet FG, Kindt R, Legendre P, Minchin PR, O’hara RB, Simpson GL, Solymos P, Stevens MH, Wagner HJ (2015). Vegan: community ecology package. R package vegan, vers. 2.2-1. Worl Agro Cent.

[65] Martinez-Arbizu P, pairwiseAdonis. Pairwise Multilevel Comparison using Adonis (R Package. version 0.3, 2019). 2019.

[66] Hartman K, van der Heijden MGA, Wittwer RA, Banerjee S, Walser J-C, Schlaeppi K (2018). Cropping practices manipulate abundance patterns of root and soil microbiome members paving the way to smart farming. Microbiome.

[67] De Cáceres M, Legendre P, Moretti M (2010). Improving indicator species analysis by combining groups of sites. Oikos.

[68] Hamilton NE, Ferry M (2018). ggtern: ternary diagrams using ggplot2. J Stat Softw.

[69] Lahti L, Shetty S. Microbiome analytics. Version 1.7.21. 55. 2019.

[70] Jiao S, Chen WM, Wei GH (2017). Biogeography and ecological diversity patterns of rare and abundant bacteria in oil-contaminated soils. Mol Ecol.

[71] Kim H, Lee KK, Jeon J, Harrie WAJ, Ly YH (2020). Domestication of Oryza species eco-evolutionarily shapes bacterial and fungal communities in rice seed. Microbiome.

[72] Toju H, Peay KG, Yamamichi M, Narisawa K, Hiruma K, Naito K, Fukuda S, Ushio M, Nakaoka S, Onoda Y, Yoshida K (2018). Core microbiomes for sustainable agroecosystems. Nat Plants.

[73] Cheng YT, Zhang L, He SY (2019). Plant-microbe interactions facing environmental challenge. Cell Host Microbe.

[74] Singh BK, Trivedi P, Egidi E, Macdonald CA, Delgado-Baquerizo M (2020). Crop microbiome and sustainable agriculture. Nat Rev Microbiol.

[75] Ahn JH, Song J, Kim BY, Kim MS, Joa JH, Weon HY (2012). Characterization of the bacterial and archaeal communities in rice field soils subjected to long-term fertilization practices. J Microbiol.

[76] Breidenbach B, Conrad R (2015). Seasonal dynamics of bacterial and archaeal methanogenic communities in flooded rice fields and effect of drainage. Front Microbiol.

[77] Lee HJ, Jeong SE, Kim PJ, Madsen EL, Jeon CO (2015). High resolution depth distribution of Bacteria, Archaea, methanotrophs, and methanogens in the bulk and rhizosphere soils of a flooded rice paddy. Front Microbiol.

[78] Hartmann M, Widmer F (2006). Community structure analyses are more sensitive to differences in soil bacterial communities than anonymous diversity indices. Appl Environ Microbiol.

[79] Wang J, Xue C, Song Y, Wang L, Huang Q, Shen Q (2016). Wheat and Rice Growth Stages and fertilization regimes Alter Soil Bacterial Community structure, but not diversity. Front Microbiol.

[80] Wu Z, Liu Q, Li Z, Cheng W, Sun J, Guo Z, Li Y, Zhou J, Meng D, Li H, Lei P (2018). Environmental factors shaping the diversity of bacterial communities that promote rice production. BMC Microbiol.

[81] Philippot L, Raaijmakers JM, Lemanceau P, van der Putten WH (2013). Going back to the roots: the microbial ecology of the rhizosphere. Nat Rev Microbiol.

[82] Aulakh MS, Wassmann R, Bueno C, Kreuzwieser J, Rennenberg H (2001). Characterization of root exudates at different growth stages of ten rice (Oryza sativa L.) cultivars. Plant Biol.

[83] Anas I, Rupela OP, Thiyagarajan TM, Uphoff N (2011). A review of studies on SRI effects on beneficial organisms in rice soil rhizospheres. Paddy and Water Environ.

[84] Meena VS, Maurya BR, Bohra JS, Verma R, Meena MD (2013). Effect of concentrate manure and nutrient levels on enzymatic activities and microbial population under submerged rice in alluvium soil of Varanasi. Crop Res.

[85] Asch F, Wopereis MCS (2001). Responses of field-grown irrigated rice cultivars to varying levels of floodwater salinity in a semi-arid environment. Field Crops Res.

[86] Fageria NK (2003). Plant tissue test for determination of Optimum Concentration and Uptake of Nitrogen at different growth stages in Lowland Rice. Commun Soil Sci Plant Anal.

[87] Fageria NK (2007). Yield physiology of rice. J plant nutr.

[88] Harbort CJ, Hashimoto M, Inoue H, Niu Y, Guan R, Rombolà AD, Kopriva S, Voges MJ, Sattely ES, Garrido-Oter R, Schulze-Lefert P (2020). Root-secreted coumarins and the microbiota interact to improve iron nutrition in Arabidopsis. Cell Host Microbe.

[89] Zhao M, Zhao J, Yuan J, Hale L, Wen T, Huang Q, Vivanco JM, Zhou J, Kowalchuk GA, Shen Q (2021). Root exudates drive soil-microbe-nutrient feedbacks in response to plant growth. Plant Cell Environ.

[90] Valentine DL (2007). Adaptations to energy stress dictate the ecology and evolution of the Archaea. Nat Rev Microbiol.

[91] Cederlund H, Wessén E, Enwall K, Jones CM, Juhanson J, Pell M, Philippot L, Hallin S (2014). Soil carbon quality and nitrogen fertilization structure bacterial communities with predictable response of major bacterial phyla. Agric Ecosyst Environ Appl.

[92] Fierer N, Lauber CL, Ramirez KS, Zaneveld J, Bradford MA, Knight R (2012). Comparative metagenomic, phylogenetic and physiological analyses of soil microbial communities across nitrogen gradients. ISME J.

[93] Zeng J, Liu X, Song L, Lin X, Zhang H, Shen C, Chu H (2016). Nitrogen fertilization directly affects soil bacterial diversity and indirectly affects bacterial community composition. Soil Biol Biochem.

[94] Ling N, Chen D, Guo H, Wei J, Bai Y, Shen Q, Hu S (2017). Differential responses of soil bacterial communities to long-term N and P inputs in a semi-arid steppe. Geoderma Front Microbiol.

[95] Orellana LH, Chee-Sanford JC, Sanford RA, Löffler FE, Konstantinidis KT (2018). Year-round shotgun metagenomes reveal stable microbial communities in agricultural soils and novel ammonia oxidizers responding to fertilization. Appl Environ Microbiol.

[96] Pathan SI, Roccotelli A, Petrovičovà B, Romeo M, Badagliacca G, Monti M, Gelsomino A (2021). Temporal dynamics of total and active prokaryotic communities in two Mediterranean orchard soils treated with solid anaerobic digestate or managed under no-tillage. Biol Fertil Soils.

[97] Coyte KZ, Schluter J, Foster KR (2015). The ecology of the microbiome: networks, competition, and stability. Science.

[98] Van der Heijden MGA, Hartmann M (2016). Networking in the plant microbiome. PLoS Biol.

[99] Fuhrman JA (2009). Microbial community structure and its functional implications. Nature.

[100] Yuan MM, Guo X, Wu L, Zhang YA, Xiao N, Ning D, Shi Z, Zhou X, Wu L, Yang Y, Tiedje JM (2021). Climate warming enhances microbial network complexity and stability. Nat Clim Chang.

[101] Agler MT, Ruhe J, Kroll S, Morhenn C, Kim ST, Weigel D, Kemen EM (2016). Microbial hub taxa link host and abiotic factors to Plant Microbiome Variation. PLOS Biol.

[102] Bapteste E, Brochier C, Boucher Y (2005). Higher-level classification of the Archaea: evolution of methanogenesis and methanogens. Archaea.

[103] Adam P, Borrel G, Brochier-Armanet C, Gribaldo S (2017). The growing tree of Archaea: new perspectives on their diversity, evolution and ecology. ISME J.

[104] Fan K, Weisenhorn P, Gilbert JA, Chu H (2018). Wheat rhizosphere harbors a less complex and more stable microbial co-occurrence pattern than bulk soil. Soil Biol Biochem.

[105] Mondav R, McCalley CK, Hodgkins SB, Frolking S, Saleska SR, Rich VI, Chanton JP, Crill PM (2017). Microbial network, phylogenetic diversity and community membership in the active layer across a permafrost thaw gradient. Environ Microbiol.

[106] Koskinen K, Pausan MR, Perras AK, Beck M, Bang C, Mora M, Schilhabel A, Schmitz R, Moissl-Eichinger C (2017). First Insights into the Diverse Human Archaeome: specific detection of Archaea in the gastrointestinal tract, lung, and nose and on skin. MBio.

[107] Moissl-Eichinger C, Pausan M, Taffner J, Berg G, Bang C, Schmitz RA (2018). Archaea are interactive components of complex microbiomes. Trends Microbiol.

[108] Samuel BS, Hansen EE, Manchester JK, Coutinho PM, Henrissat B, Fulton R, Latreille P, Kim K, Wilson RK, Gordon JI (2007). Genomic and metabolic adaptations of Methanobrevibacter smithii to the human gut. P Natl Acad Sci USA.

[109] Bang C, Schmitz RA (2015). Archaea associated with human surfaces: not to be underestimated. FEMS Microbiol Rev.

[110] Bang C, Dagan T, Deines P, Dubilier N, Duschl WJ, Fraune S, Hentschel U, Hirt H, Hülter N, Lachnit T, Picazo D, Pita L, Pogoreutz C, Rädecker N, Saad MM, Schmitz RA, Schulenburg H, Voolstra CR, Weiland-Bräuer N, Ziegler M, Bosch TCG (2018). Metaorganisms in extreme environments: do microbes play a role in organismal adaptation?. Zool.

[111] Leigh JA (2000). Nitrogen fixation in methanogens: the archaeal perspective. Curr Issues Mol Biol.

[112] Dixon R, Kahn D (2004). Genetic regulation of biological nitrogen fixation. Nat Rev Microbiol.

[113] Dekas AE, Poretsky RS, Orphan VJ (2009). Deep-sea archaea fix and share nitrogen in methane-consuming microbial consortia. Sci.

[114] Morris BE, Henneberger R, Huber H, Moissl-Eichinger C (2013). Microbial syntrophy: interaction for the common good. FEMS Microbiol Rev.

[115] Guo L, Zheng S, Cao C, Li C (2016). Tillage practices and straw-returning methods affect topsoil bacterial community and organic C under a rice-wheat cropping system in central China. Sci Rep.

[116] Wang YH, Yu ZH, Li YS, Wang GH, Liu JJ, Liu JD, Liu XB, Jin J (2017). Microbial association with the dynamics of particulate organic carbon in response to the amendment of elevated CO2-derived wheat residue into a Mollisol. Sci Total Environ.

[117] Xu L, Han Y, Yi M, Yi H, Guo E, Zhang A (2019). Shift of millet rhizosphere bacterial community during the maturation of parent soil revealed by 16S rDNA high-throughput sequencing. Appl soil Ecol.

[118] Yang Y, Wang N, Guo X, Zhang Y, Ye B (2017). Comparative analysis of bacterial community structure in the rhizosphere of maize by high throughput pyrosequencing. PLoS ONE.

[119] Cui J, Li Y, Wang C, Kim KS, Wang T, Liu S (2018). Characteristics of the rhizosphere bacterial community across different cultivation years in saline-alkaline paddy soils of Songnen Plain of China. Can J Microbiol.

[120] Shen Z, Wang D, Ruan Y, Xue C, Zhang J, Li R, Shen Q. Deep 16S rRNA Pyrosequencing Reveals a Bacterial Community Associated with Banana Fusarium Wilt Disease Suppression Induced by Bio-Organic Fertilizer Application. PloS One. 2014;9(5):e98420. pmid:24871319. 10.1371/journal.pone.0098420. 10.1371/journal.pone.0098420 PMC4037203 24871319

[121] Shen Z, Wang B, Zhu J, Hu H, Tao C, Ou Y, Deng X, Ling N, Li R, Shen Q (2019). Lime and ammonium carbonate fumigation coupled with bio-organic fertilizer application steered banana rhizosphere to assemble a unique microbiome against Panama disease. Microb Biotechnol.

[122] Li F, Chen L, Zhang J, Yin J, Huang S (2017). Bacterial Community structure after long-term Organic and Inorganic fertilization reveals important Associations between Soil Nutrients and specific Taxa involved in nutrient transformations. Front Microbiol.

[123] Xun W, Liu Y, Li W, Ren Y, Xiong W, Xu Z, Zhang N, Miao Y, Shen Q, Zhang R (2021). Specialized metabolic functions of keystone taxa sustain soil microbiome stability. Microbiome.

[124] Wang S, Ding L, Liu W, Wang J, Qian Y (2021). Effect of plastic mulching on soil carbon and nitrogen cycling-related bacterial community structure and function in a dryland spring maize field. Agriculture.

[125] Guo J, Cheng J, Li B, Wang J, Chu P (2019). Performance and microbial community in the biocathode of microbial fuel cells under different dissolved oxygen concentrations. J Electroanal Chem.

[126] Xia Z, Wang Q, She Z, Gao M, Zhao Y, Guo L, Jin C (2019). Nitrogen removal pathway and dynamics of microbial community with the increase of salinity in simultaneous nitrification and denitrification process. Sci Total Environ.

