# Supplementary material for: Rice developmental stages modulate rhizosphere bacteria and archaea co-occurrence and sensitivity to long-term inorganic fertilization in a West African Sahelian agro-ecosystem
Source: Environ Microbiome. 2023 May 17;18:42. doi: 10.1186/s40793-023-00500-1 (PMC10193678; doi:10.1186/s40793-023-00500-1)
Supplement: Supplementary file 1 — Supplementary Material 1 [file 40793_2023_500_MOESM1_ESM.docx]

**Rice developmental stages modulate rhizosphere bacteria and archaea co-occurrence and sensitivity to long-term inorganic fertilization in a West African Sahelian agro-ecosystem**

**Additional file 1:**

**Table S1:** Results from ANOVA of the linear mixed effects (LME) model testing the effect of long-term fertilization and developmental stage on Shannon index of bacterial and archaeal community inhabiting the rice rhizosphere. R^2^m (marginal r squared) represents the variance explained by the fixed factors, R^2^c (conditional r squared) the variance explained by the both fixed and random factors. The model was run with fertilization (Fer), sampling time-point (ST) nested within developmental stage (DS) and their interaction as fixed factors and plot block as random factor

|  | Shannon index | | | | |
| --- | --- | --- | --- | --- | --- |
|  | df | Chisq | Pr(>Chisq) | R^2^m | R^2^c |
| Bacteria | | | | | |
| Fer | 2 | 6.627 | 0.037 | 0.364 | 0.553 |
| DS | 2 | 0.035 | 0.983 |  |  |
| Fer:DS | 4 | 12.713 | 0.013 |  |  |
| Fer:DS:ST | 9 | 10.567 | 0.307 |  |  |
| Archaea | | | | | |
| Fer | 2 | 14.773 | 0.001 | 0.451 | 0.545 |
| DS | 2 | 20.284 | 3.939e-05 |  |  |
| Fer:DS | 4 | 4.617 | 0.329 |  |  |
| Fer:DS:ST | 9 | 11.345 | 0.253 |  |  |

**Table S2:** Results of PERMANOVA based on Bray-Curtis distance testing the effects of long-term fertilization (Fer) and developmental stage (DS) on bacterial and archaeal community inhabiting the rice rhizosphere. The model was run with sampling time-point (ST) nested within developmental stage and 9999 permutations constrained within plot block

|  | df | SumOfSqs | R2 | F | Pr(>F) |
| --- | --- | --- | --- | --- | --- |
| Bacteria | | | | | |
| Fer | 2 | 1.295 | 0.133 | 3.760 | 1e-04 |
| DS | 2 | 0.790 | 0.081 | 2.293 | 1e-04 |
| Fer:DS | 4 | 0.617 | 0.063 | 0.896 | 0.376 |
| Fer:DS:ST | 9 | 1.208 | 0.124 | 0.779 | 0.978 |
| Residual | 34 | 5.854 | 0.600 |  |  |
| Total | 51 | 9.763 | 1 |  |  |
| Archaea | | | | | |
| Fer | 2 | 1.469 | 0.239 | 7.039 | 1e-04 |
| DS | 2 | 0.286 | 0.047 | 1.371 | 0.023 |
| Fer:DS | 4 | 0.258 | 0.042 | 0.619 | 0.910 |
| Fer:DS:ST | 9 | 0.573 | 0.093 | 0.610 | 0.990 |
| Residual | 34 | 3.549 | 0.578 |  |  |
| Total | 51 | 6.136 | 1 |  |  |

**Table S3:** Results from ANOVA testing the effects of long-term fertilization (NPK-fertilization (NPK), N-fertilization (N), and non-fertilization control (UF)) on soil properties and Mantel test based on Spearman’s correlations testing the relationship between microbial communities and soil properties. Different letters in the pairwise comparisons indicate significant differences at p<0.05

| Soil properties | | pH_H2O_ | pH_KCl_ | Conductivity (µS/cm) | Salinity (%) | N(NO_3_)  (mg/kg) | N(NH_4_)  (mg/kg) | TN  (%) | TC (%) | C/N | TP (mg/kg) | AP (mg/kg) |
| --- | --- | --- | --- | --- | --- | --- | --- | --- | --- | --- | --- | --- |
| ANOVA | | | | | | | | | | | | |
| Fertilization | F | 15.840 | 8.211 | 4.620 | 2.333 | 0.984 | 0.614 | 0.524 | 0.523 | 0.573 | 25.43 | 8.53 |
|  | P | 0.004 | 0.019 | 0.061 | 0.178 | 0.427 | 0.572 | 0.617 | 0.618 | 0.592 | 0.001 | 0.017 |
| Pairwise Comparisons | | | | | | | | | | | | |
| Fertilizer treatments | UF | 6.312 b | 5.409 b | 716.333 a | 0.367 a | 6.548 a | 6.819 a | 0.070 a | 0.785 a | 11.119 a | 112.467 b | 0.881 b |
|  | N | 6.611 a | 5.367 b | 370.667 a | 0.267 a | 5.871 a | 8.322 a | 0.071 a | 0.817 a | 11.538 a | 132.760 b | 2.461 ab |
|  | NPK | 6.817 a | 5.767 a | 562.333 a | 0.333 a | 7.143 a | 6.922 a | 0.079 a | 0.876 a | 11.160 a | 220.300 a | 7.225 a |
| Mantel test (correlations between microbial communities and soil properties) | | | | | | | | | | | | |
| Bacteria | R | 0.333 | 0.444 | 0.293 | 0.213 | 0.287 | 0.420 | 0.409 | 0.442 | 0.146 | 0.485 | 0.449 |
|  | P | 0.0001 | 0.0001 | 0.0001 | 0.0002 | 0.0001 | 0.0001 | 0.0001 | 0.0001 | 0.005 | 0.0001 | 0.0001 |
| Archaea | R | 0.410 | 0.386 | 0.254 | 0.109 | 0.151 | 0.454 | 0.324 | 0.328 | -0.009 | 0.427 | 0.285 |
|  | P | 0.0001 | 0.0001 | 0.0001 | 0.023 | 0.001 | 0.0001 | 0.0001 | 0.0001 | 0.547 | 0.0001 | 0.0002 |

N indicates nitrogen; C, carbon; P, phosphorus; TN, total nitrogen; TC, total carbon; TP, total phosphorus; AP, assimilable phosphorus

**Table S4:** Results of PERMANOVA based on Bray-Curtis distance testing the effects of long-term fertilization (NPK-fertilization (NPK), N-fertilization (N), and non-fertilization control (UF)) and sampling time-point (ST) on bacterial and archaeal community inhabiting the rice rhizosphere at different developmental stages, and the results of pairwise comparisons and multivariate homogeneity of groups dispersions (BETADISPER). Fer refers to fertilization and the models were run with 9999 permutations constrained within plot block

|  | Bacteria | | | | | | Archaea | | | | | |
| --- | --- | --- | --- | --- | --- | --- | --- | --- | --- | --- | --- | --- |
|  | Tillering | | Panicle initiation | | Booting | | Tillering | | Panicle initiation | | Booting | |
| Permutation test for adonis (PERMANOVA) | | | | | | | | | | | | |
|  | F | R2 | F | R2 | F | R2 | F | R2 | F | R2 | F | R2 |
| ST | 1.029 | 0.055 | 0.693 | 0.037 | 1.141 | 0.063 | 0.955 | 0.045 | 0.603 | 0.029 | 0.831 | 0.045 |
| Fer | 1.810*** | 0.194 | 2.425*** | 0.262 | 1.940*** | 0.215 | 3.211*** | 0.300 | 3.699*** | 0.352 | 2.397*** | 0.257 |
| Pairwise fertilization regime comparisons (with FDR adjusted p-value) | | | | | | | | | | | | |
| UF vs NPK | 1.889* | 0.159 | 2.387** | 0.210 | 1.854** | 0.156 | 4.302** | 0.301 | 4.992** | 0.357 | 3.470** | 0.258 |
| N vs UF | 1.319 | 0.117 | 1.669* | 0.143 | 1.929** | 0.177 | 2.234* | 0.183 | 2.010 | 0.167 | 1.960* | 0.179 |
| NPK vs N | 2.248** | 0.184 | 3.479** | 0.279 | 1.977** | 0.180 | 3.193** | 0.242 | 4.572** | 0.337 | 1.926* | 0.176 |
| Multivariate homogeneity of groups dispersions (betadisper) | | | | | | | | | | | | |
|  | F | Pr(>F) | F | Pr(>F) | F | Pr(>F) | F | Pr(>F) | F | Pr(>F) | F | Pr(>F) |
| Fer | 1.138 | 0.111 | 0.461 | 0.572 | 0.761 | 0.364 | 4.024 | 0.028* | 3.237 | 0.009** | 2.022 | 0.081 |

Stars indicate significant effects: * = *P* < 0.05, ** = *P* < 0.01, *** = *P* < 0.001

**Table S5:** Properties of core bacteria and archaea co-occurrence networks at different rice developmental stages (tillering, panicle initiation, booting), with the number of bacterial and archaeal nodes, their connections (edges: Bac-Bac, Arc-Arc, Bac-Arc), and the number of long-term inorganic fertilization sensitive (*lifs*) OTUs, with in parentheses the number of potential hub OTUs

| Core bacteria and archaea co-occurrence meta-networks | | Tillering | Panicle initiation | Booting |
| --- | --- | --- | --- | --- |
| Network nodes | Total number of nodes | 814 | 1040 | 910 |
|  | Number of bacteria nodes | 391 | 927 | 484 |
|  | Number of archaea nodes | 423 | 113 | 426 |
| Network edges | Total number of edges | 1031 | 1179 | 965 |
|  | Number of bacteria - bacteria connections | 290 | 457 | 346 |
|  | Number of archaea - archaea connections | 606 | 473 | 526 |
|  | Number of bacteria - archaea connections | 135 | 249 | 93 |
| Network topological features | Average degree | 2.533 | 2.267 | 2.121 |
|  | Network diameter | 22 | 16 | 15 |
|  | Average path length | 8.185 | 5.829 | 4.551 |
|  | Graph density | 0.003 | 0.002 | 0.002 |
|  | Modularity | 0.917 | 0.908 | 0.965 |
|  | Clustering coefficient | 0.475 | 0.443 | 0.533 |
|  | Weakly connected components | 179 | 287 | 254 |
| Number of positive correlations | | 890 | 920 | 861 |
| Number of negative correlations | | 141 | 259 | 104 |
| Number of potential hub OTUs of bacteria | | 0 | 2 | 0 |
| Number of potential hub OTUs of archaea | | 2 | 8 | 6 |
| Number of *lifs* bacterial OTUs | | 56 (0) | 184 (2) | 90 (0) |
| Number of *lifs* archaeal OTUs | | 97 (0) | 113 (5) | 89 (5) |

**Table S6:** The potential hub OTUs identified from the microbial inter-kingdom networks obtained at tillering (Tl), panicle initiation (Pi) and booting stage (Bt) of field-grown rice. *Lifs* OTUs represent those sensitive to long-term inorganic fertilization

| OTU ID | Kingdom | Phylum | Class | Order | Family | Genus | *Lifs* OTU | Tl | Pi | Bt |
| --- | --- | --- | --- | --- | --- | --- | --- | --- | --- | --- |
| OTU27502 | Archaea | Euryarchaeota | Methanomicrobia | Methanosarcinales | Methanosaetaceae | Methanosaeta | No | x |  |  |
| OTU31817 | Archaea | Euryarchaeota | Methanobacteria | Methanobacteriales | Methanobacteriaceae | Methanobacterium | No | x |  |  |
| OTU29189 | Archaea | Euryarchaeota | Methanobacteria | Methanobacteriales | Methanobacteriaceae | Methanobacterium | Yes |  | x |  |
| OTU32729 | Archaea | Euryarchaeota | Methanobacteria | Methanobacteriales | Methanobacteriaceae | Methanobacterium | Yes |  | x |  |
| OTU33199 | Archaea | Euryarchaeota | Methanobacteria | Methanobacteriales | Methanobacteriaceae | Methanobacterium | Yes |  | x |  |
| OTU33593 | Archaea | Euryarchaeota | Methanobacteria | Methanobacteriales | Methanobacteriaceae | Methanobacterium | Yes |  | x |  |
| OTU31230 | Archaea | Euryarchaeota | Methanomicrobia | Methanosarcinales | Methanosaetaceae | Methanosaeta | Yes |  | x |  |
| OTU32999 | Archaea | Euryarchaeota | Methanomicrobia | Methanosarcinales | Methanosaetaceae | Methanosaeta | No |  | x |  |
| OTU33541 | Archaea | Euryarchaeota | Methanomicrobia | Methanomicrobiales | Methanoregulaceae | Methanosphaerula | No |  | x |  |
| OTU153 | Bacteria | Gemmatimonadetes | Gemmatimonadetes | Gemmatimonadales | Gemmatimonadaceae | Gemmatimonas | Yes |  | x |  |
| OTU363 | Bacteria | Planctomycetes | Planctomycetia | Planctomycetales | Planctomycetaceae | Pirellula | Yes |  | x |  |
| OTU32026 | Archaea | Euryarchaeota | Methanomicrobia | Methanosarcinales | Methanosaetaceae | Methanosaeta | No |  |  | x |
| OTU32178 | Archaea | Euryarchaeota | Methanomicrobia | Methanosarcinales | Methanosaetaceae | Methanosaeta | Yes |  |  | x |
| OTU32278 | Archaea | Euryarchaeota | Methanomicrobia | Methanosarcinales | Methanosaetaceae | Methanosaeta | Yes |  |  | x |
| OTU32636 | Archaea | Euryarchaeota | Methanomicrobia | Methanosarcinales | Methanosaetaceae | Methanosaeta | Yes |  |  | x |
| OTU33489 | Archaea | Euryarchaeota | Methanomicrobia | Methanosarcinales | Methanosaetaceae | Methanosaeta | Yes |  |  | x |
| OTU32957 | Archaea | Euryarchaeota | Methanobacteria | Methanobacteriales | Methanobacteriaceae | Methanobacterium | Yes |  |  | x |
