# Supplementary material for: Rice developmental stages modulate rhizosphere bacteria and archaea co-occurrence and sensitivity to long-term inorganic fertilization in a West African Sahelian agro-ecosystem
Source: Environ Microbiome. 2023 May 17;18:42. doi: 10.1186/s40793-023-00500-1 (PMC10193678; doi:10.1186/s40793-023-00500-1)
Supplement: Supplementary file 2 — Supplementary Material 2 [file 40793_2023_500_MOESM2_ESM.docx]

**Rice developmental stages modulate rhizosphere bacteria and archaea co-occurrence and sensitivity to long-term inorganic fertilization in a West African Sahelian agro-ecosystem**

**Additional file 2:**


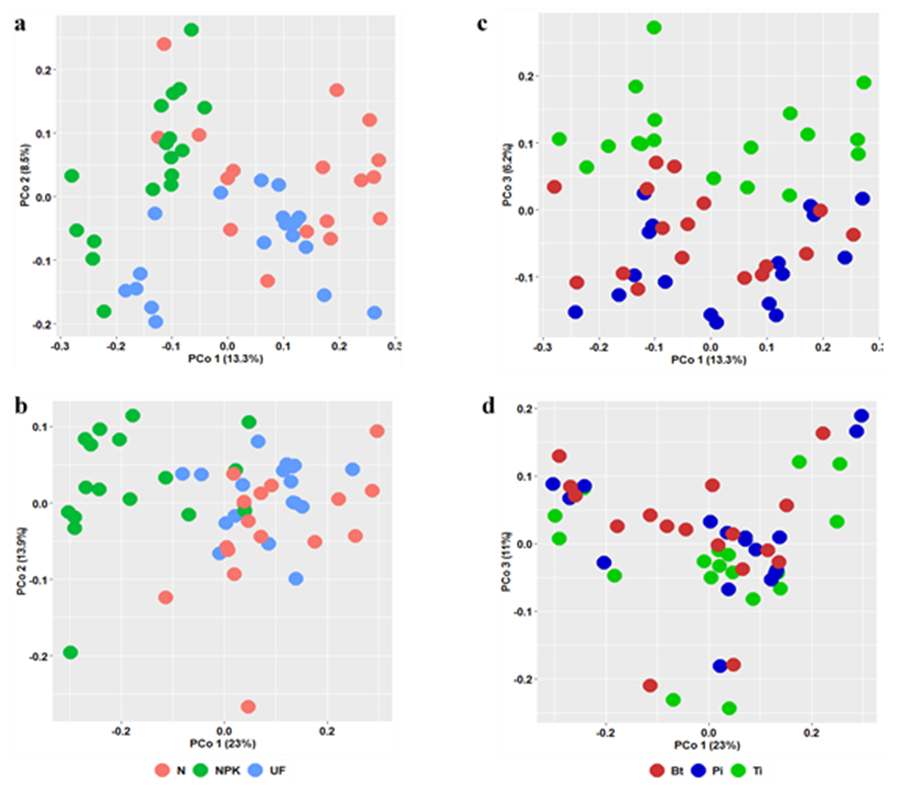


**Fig. S1:** Unconstrained PCoA ordinations of bacteria (a and c) and archaea (b and d) inhabiting the rice rhizosphere. Panels a and b show principal coordinate PCo 1 versus PCo 2, with the points colored by fertilization (NPK-fertilization (NPK), N-fertilization (N), and non-fertilization control (UF)), and panels c and d show principal coordinate PCo 1 versus PCo 3, with the points colored by developmental stage (tillering (Ti), panicle initiation (Pi) and booting stage (Bt)). The explained fraction of total variation in each community is given on each axis


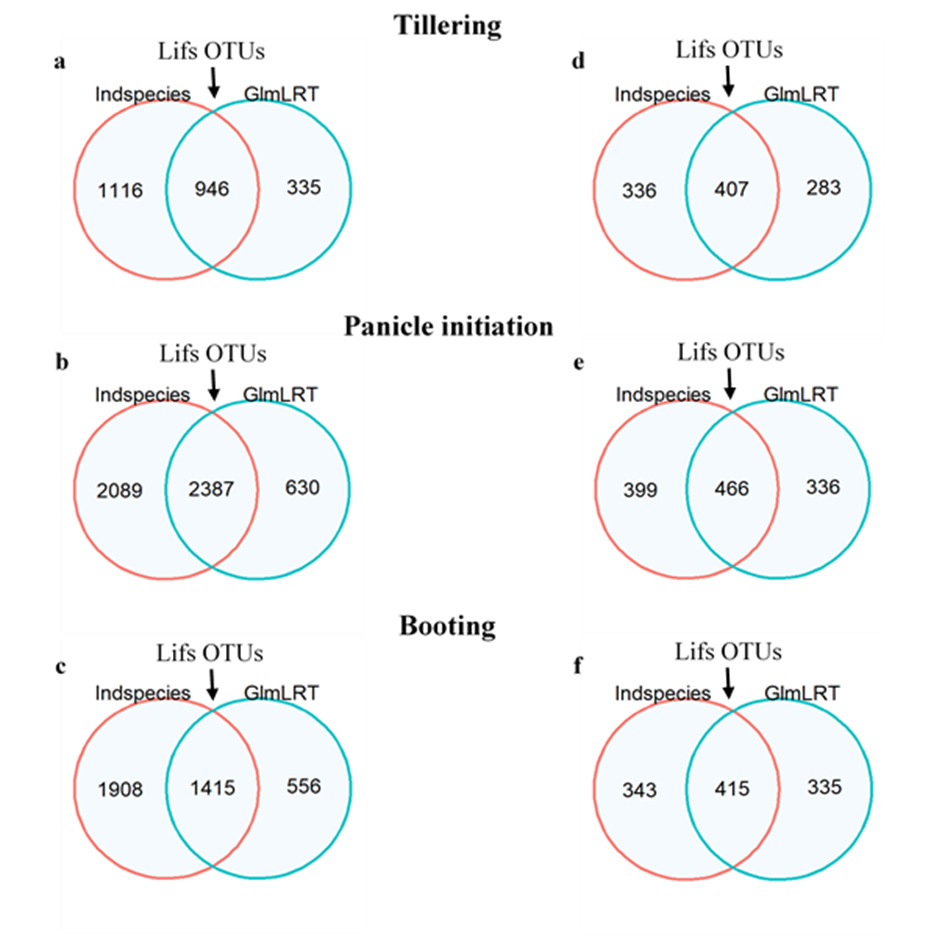


**Fig. S2:** Determining the long-term inorganic fertilization sensitive (*lifs*) OTUs of bacteria (a, b and c) and archaea (d, e and f) inhabiting the rice rhizosphere at tillering, panicle initiation and booting stage in unfertilized (UF), N-fertilized (N) and NPK-fertilized (NPK) plots. Red cycles indicate the number of OTUs responding to long-term inorganic fertilization identified by indicator species analysis and blue cycles those identified by edgeR. The *lifs* OTUs are those identified by both methods


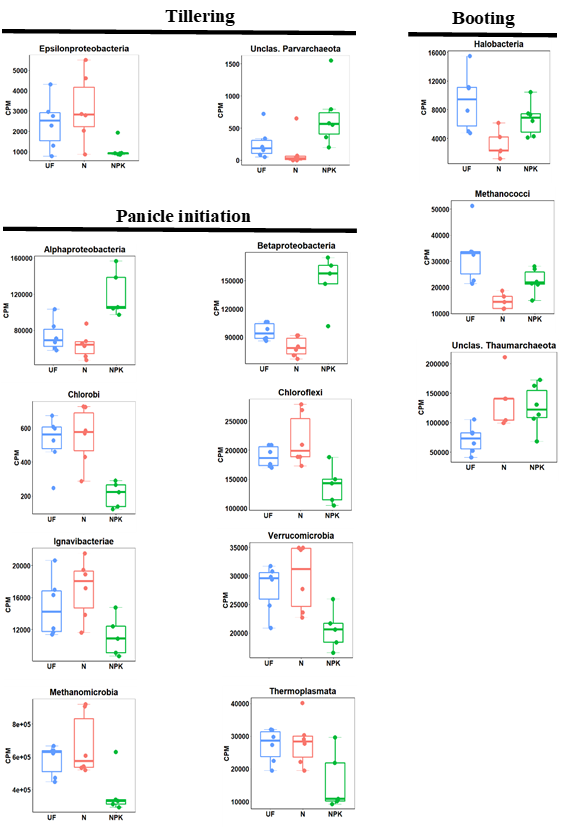


**Fig. S3:** Relative abundances (counts per million, CPM) of bacterial phyla (classes for Proteobacteria) and archaeal classes (classified and unclassified (Unclas.)) identified as sensitive to long-term inorganic fertilization at tillering, panicle initiation and booting stage of rice grown in unfertilized (UF), N-fertilized (N) and NPK-fertilized (NPK) plots


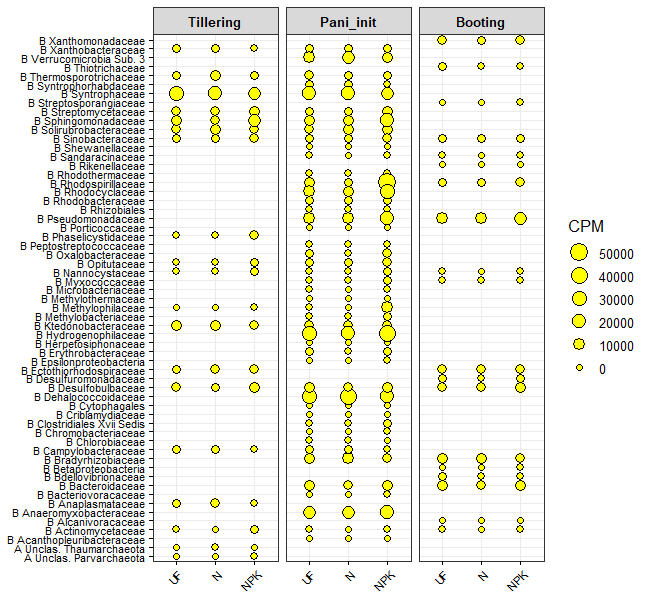


**Fig. S4:** Rhizosphere bacterial and archaeal families identified as sensitive to long-term inorganic fertilization at tillering, panicle initiation (Pani_init) and booting stage of rice grown in unfertilized (UF), N-fertilized (N) and NPK-fertilized (NPK) plots. The mean relative abundances of each family (classified or unclassified (Unclas.)) in each plot at each developmental stage are given in counts per million (CPM). The letters A and B associated with family names indicate archaeal and bacterial kingdom, respectively


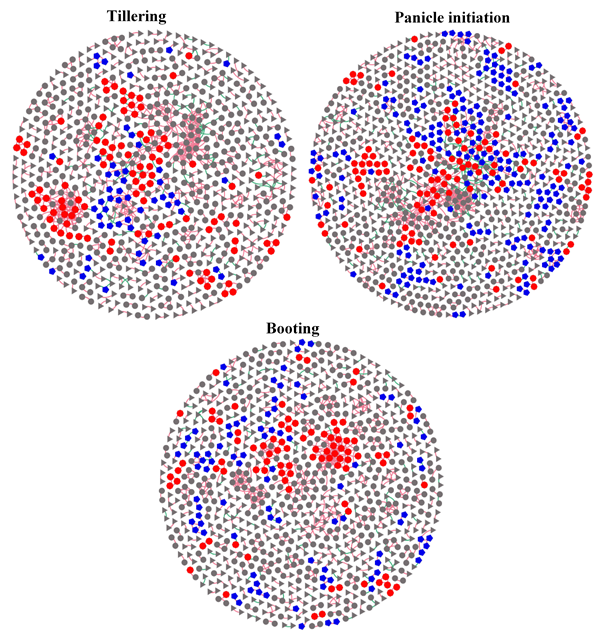


**Fig. S5:** Co-occurrence-based inter-kingdom networks of the core microbiome taxa inhabiting the rice rhizosphere at tillering, panicle initiation and booting stages. Each node corresponds to an OTU, and edges correspond to either positive (pink) or negative (green) correlations inferred from OTU abundances. Circles represent archaeal OTUs, and triangles bacterial OTUs. Red asterisks represent long-term inorganic fertilization sensitive (*lifs*) OTUs of archaea and blue pentagons bacterial *lifs* OTUs. The shared potential hub OTUs between developmental stages are presented in Table S6
